# Epigenome-wide association study of kidney function identifies trans-ethnic and ethnic-specific loci

**DOI:** 10.1186/s13073-021-00877-z

**Published:** 2021-04-30

**Authors:** Charles E. Breeze, Anna Batorsky, Mi Kyeong Lee, Mindy D. Szeto, Xiaoguang Xu, Daniel L. McCartney, Rong Jiang, Amit Patki, Holly J. Kramer, James M. Eales, Laura Raffield, Leslie Lange, Ethan Lange, Peter Durda, Yongmei Liu, Russ P. Tracy, David Van Den Berg, NHLBI Trans-Omics for Precision Medicine (TOPMed) Consortium, TOPMed MESA Multi-Omics Working Group, Kathryn L. Evans, William E. Kraus, Svati Shah, Hermant K. Tiwari, Lifang Hou, Eric A. Whitsel, Xiao Jiang, Fadi J. Charchar, Andrea A. Baccarelli, Stephen S. Rich, Andrew P. Morris, Marguerite R. Irvin, Donna K. Arnett, Elizabeth R. Hauser, Jerome I. Rotter, Adolfo Correa, Caroline Hayward, Steve Horvath, Riccardo E. Marioni, Maciej Tomaszewski, Stephan Beck, Sonja I. Berndt, Stephanie J. London, Josyf C. Mychaleckyj, Nora Franceschini

**Affiliations:** 1 Division of Cancer Epidemiology and Genetics, National Cancer Institute, National Institutes of Health, Department Health and Human Services, Bethesda, MD USA; 2 UCL Cancer Institute, University College London, London, WC1E 6BT UK; 3 Altius Institute for Biomedical Sciences, Seattle, WA 98121 USA; 4 Department of Biostatistics, University of North Carolina, Chapel Hill, NC 27516 USA; 5 Epidemiology Branch, National Institute of Environmental Health Sciences, National Institutes of Health, Department of Health and Human Services, Research Triangle Park, NC 27709 USA; 6 Division of Biomedical Informatics and Personalized Medicine, University of Colorado Anschutz Medical Campus, Aurora, CO USA; 7 Division of Cardiovascular Sciences, Faculty of Biology, Medicine and Health, University of Manchester, Manchester, UK; 8 Centre for Genomic and Experimental Medicine, Institute of Genetics and Molecular Medicine, University of Edinburgh, Crewe Road, Edinburgh, EH4 2XU UK; 9 Department of Psychiatry and Behavioral Sciences, Duke University Medical Center, Durham, NC 27701 USA; 10 Department of Biostatistics, University of Alabama, Birmingham, AL USA; 11 Department of Public Health Sciences and Medicine, Loyola University Chicago, Maywood, IL USA; 12 Division of Nephrology and Hypertension, Loyola University Chicago, Maywood, IL USA; 13 Department of Genetics, University of North Carolina, Chapel Hill, NC USA; 14 Department of Pathology & Laboratory Medicine, Larner College of Medicine, University of Vermont, Burlington, VT USA; 15 Duke Molecular Physiology Institute, Duke University Medical Center, Durham, NC USA; 16 Department of Biochemistry, Larner College of Medicine, University of Vermont, Burlington, VT USA; 17 Center for Genetic Epidemiology, Department of Preventive Medicine, Keck School of Medicine of USC, University of Southern California, Los Angeles, CA USA; 18 Division of Cardiology, Department of Medicine, School of Medicine, Duke University, Durham, NC USA; 19 Department of Preventive Medicine, Northwestern University Feinberg School of Medicine, Chicago, IL USA; 20 Center for Global Oncology, Institute of Global Health, Northwestern University Feinberg School of Medicine, Chicago, IL USA; 21 Department of Epidemiology, University of North Carolina, Chapel Hill, NC USA; 22 Department of Medicine, University of North Carolina, Chapel Hill, NC 27599 USA; 23 School of Health and Life Sciences, Federation University Australia, Ballarat, VIC Australia; 24 Department of Physiology, University of Melbourne, Parkville, VIC Australia; 25 Department of Cardiovascular Sciences, University of Leicester, Leicester, UK; 26 Laboratory of Environmental Epigenetics, Departments of Environmental Health Sciences and Epidemiology, Columbia University Mailman School of Public Health, New York, NY USA; 27 Center for Public Health Genomics, University of Virginia, Charlottesville, VA USA; 28 Centre for Genetics and Genomics Versus Arthritis, Centre for Musculoskeletal Research, The University of Manchester, Manchester, UK; 29 Department of Epidemiology, University of Alabama at Birmingham, Birmingham, AL USA; 30 College of Public Health, University of Kentucky, Lexington, KY USA; 31 Durham VA Health System, Durham, NC 27705 USA; 32 The Institute for Translational Genomics and Population Sciences, Department of Pediatrics, The Lundquist Institute for Biomedical Innovation at Harbor-UCLA Medical Center, Torrance, CA USA; 33 Department of Medicine, University of Mississippi Medical Center, Jackson, MS USA; 34 MRC Human Genetics Unit, Institute of Genetics and Cancer, University of Edinburgh, Crewe Road, Edinburgh, EH4 2XU UK; 35 Department of Human Genetics, David Geffen School of Medicine, University of California Los Angeles, Los Angeles, CA 90095 USA; 36 Department of Biostatistics, Fielding School of Public Health, University of California Los Angeles, Los Angeles, CA 90095 USA; 37 Manchester Heart Centre and Manchester Academic Health Science Centre, Manchester University NHS Foundation Trust, Manchester, UK

**Keywords:** Epigenetic, Kidney function, Gene regulation, Kidney development, DNA methylation

## Abstract

**Background:**

DNA methylation (DNAm) is associated with gene regulation and estimated glomerular filtration rate (eGFR), a measure of kidney function. Decreased eGFR is more common among US Hispanics and African Americans. The causes for this are poorly understood. We aimed to identify trans-ethnic and ethnic-specific differentially methylated positions (DMPs) associated with eGFR using an agnostic, genome-wide approach.

**Methods:**

The study included up to 5428 participants from multi-ethnic studies for discovery and 8109 participants for replication. We tested the associations between whole blood DNAm and eGFR using beta values from Illumina 450K or EPIC arrays. Ethnicity-stratified analyses were performed using linear mixed models adjusting for age, sex, smoking, and study-specific and technical variables. Summary results were meta-analyzed within and across ethnicities. Findings were assessed using integrative epigenomics methods and pathway analyses.

**Results:**

We identified 93 DMPs associated with eGFR at an FDR of 0.05 and replicated 13 and 1 DMPs across independent samples in trans-ethnic and African American meta-analyses, respectively. The study also validated 6 previously published DMPs. Identified DMPs showed significant overlap enrichment with DNase I hypersensitive sites in kidney tissue, sites associated with the expression of proximal genes, and transcription factor motifs and pathways associated with kidney tissue and kidney development.

**Conclusions:**

We uncovered trans-ethnic and ethnic-specific DMPs associated with eGFR, including DMPs enriched in regulatory elements in kidney tissue and pathways related to kidney development. These findings shed light on epigenetic mechanisms associated with kidney function, bridging the gap between population-specific eGFR-associated DNAm and tissue-specific regulatory context.

## Background

The kidney has a central role in body homeostasis through the regulation of blood pressure, fluid, and electrolytes and by removing endogenous and exogenous toxins. Reduced kidney function measured using estimated glomerular filtration rate (eGFR) defines chronic kidney disease (CKD). CKD affects 14.5% of the adult US population and is a leading cause of death and disability [1, 2]. CKD has a high burden among non-European US ethnic groups, but mechanisms for this health disparity are poorly understood [3]. A better understanding of the mechanisms influencing kidney function may provide insights into CKD occurrence and risk.

Complex interactions between genetic, lifestyle, and environmental exposures likely contribute to the observed eGFR variation across populations. DNA sequence variation accounts for 7.6% of the estimated heritability of eGFR in trans-ethnic genome-wide association studies (GWAS) [4]. Epigenetic modifications of the genome such as DNA methylation (DNAm) are heritable and contribute to gene regulation. DNAm consists of the addition of a methyl group to cytosines, typically at cytosine-guanine dinucleotides (CpG sites). DNAm is influenced by lifetime exposures and may provide clues on ethnic-specific differences influencing eGFR. Differential DNAm at CpG sites can be studied using microarrays with reasonable genome-wide coverage through epigenome-wide association studies (EWAS) [5, 6].

Recent EWAS in whole blood have identified differentially methylated positions (DMPs) associated with blood pressure and eGFR, and disease states such as CKD and rapid decline in eGFR [7–11]. Early studies had modest sample sizes (40 to 407 individuals) or were limited to a single ethnic group [7–9]. A large EWAS performed separate discovery analyses within the Atherosclerosis Risk in Communities (ARIC, 2264 African Americans) and the Framingham Heart Study (FHS, 2395 white participants) followed by cross-replication of findings [11]. The study identified 19 DMPs for eGFR or CKD. Overall, these studies support a role for DNAm in CKD-related traits. However, previous studies did not account for important potential confounders such as smoking status and cumulative exposure in the discovery group, which have widespread effects on DNAm patterns [12] and are risk factors for CKD, nor did they assess DNAm at CpG sites across multiple ethnic groups during discovery.

The main aim of this study is to identify DNAm patterns associated with eGFR in multi-ethnic studies using data from European/European American (EA), African American (AA), and Hispanic/Latino (H/L) participants. We performed both trans-ethnic and ethnic-specific EWAS using whole blood-based Illumina DNAm data assayed in participants of the Women’s Health Initiative (WHI), the Multi-Ethnic Atherosclerosis Study (MESA), and the Jackson Heart Study (JHS). We replicated our findings in the HyperGEN, Generation Scotland, and CATHGEN studies, in addition to analyzing results via look-ups in a published study [11]. We identified DMPs associated with eGFR in trans-ethnic and ethnic-specific analyses, and provided supporting evidence for the contribution of identified DMPs to kidney function and development using in silico approaches and human kidney tissue-specific data.

## Methods

### Study design and populations

Our study design included a discovery step comprising three population-based studies (WHI, MESA, and JHS) and three replication studies (HyperGEN, Generation Scotland, and CATHGEN) (Additional file 1: Fig. S1). WHI is a study of postmenopausal women (aged 50–79 years), comprising 161,808 women recruited from 40 US clinical centers who participated in an observational study or in clinical trials during 1993–1998 as previously described [13–16]. MESA is a multi-ethnic study of subclinical cardiovascular disease and risk factors for cardiovascular disease [17], consisting of 6814 asymptomatic men and women aged 45–84 (38% EA, 28% AA, 22% H/L, and 12% Asian) recruited from six field centers across the USA and examined in 2000–2002, followed by four subsequent examination periods. JHS is a study of cardiovascular disease and its risk factors in AA, comprising 5306 African Americans aged 21 to 94 years recruited from the Jackson, MS, metropolitan area from 2000 to 2004, with four follow-up exams [18]. HyperGEN is a family-based study with a sib-pair design. Hypertensive African American sibships were recruited from Forsyth County, NC, and from the community-at-large in Birmingham, AL, from 1995 to 2000 [19]. Generation Scotland is a family-based and population-based study consisting of 23,690 European participants recruited via general medical practices across Scotland between the years 2006 and 2011 [20]. CATHGEN is a biorepository of clinical samples from a prospectively collected clinical cohort of individuals undergoing cardiac catheterization at Duke University [21]. Both discovery and replication included multi-ethnic studies. Study descriptions are shown in Additional file 2: Supplementary Methods.

### Phenotypes

Serum creatinine-based eGFR was estimated using the Chronic Kidney Disease Epidemiology equation which includes age, sex, and a constant for AA [22].

### Epigenetic data and quality control

Briefly, preprocessing included removal of probes with detection *p* values > 0.01 in > 10% of samples and samples with detection *p* values > 0.01 in > 1% of probes. Beta values were normalized using beta-mixture quantile (BMIQ) normalization method (WHI-BAA and WHI-EMPC) [23], the normal-exponential out-of-band (NOOB) preprocessing method (JHS, MESA, CATHGEN) [24], Subset Quantile Normalization (SQN) (CATHGEN) [25], or dasen (Generation Scotland) [26]. Batch effect correction was performed using ComBat [27] or adjusting batch as a covariate. CpGs overlapping with the list of potentially polymorphic sites in the relevant ethnic group and cross-reactive probes were removed [28]. Cell proportions were estimated using the reference-based Houseman method for whole blood [29]. To adjust for population structure, principal components (PCs) or ethnic-informative markers were obtained from the genome-wide genotype data available using standard methods [30]. DNAm sites were annotated to include chromosome, position, UCSC gene names, relationship to CpG islands, location in gene enhancer regions, and DNase I hypersensitive sites (DHSs) using Illumina’s annotation file [31]. Detailed methods for MESA, which have not been previously published, are included in Additional file 2: Supplementary Methods.

### EWAS

Methylation betas were used as predictors for eGFR in ethnic-stratified analyses, adjusting for age, sex, smoking history (current, past, and no smoking, and pack-years), 4 to 10 principal components, cell type composition, and study-specific covariates. DMPs were modeled by fitting robust linear models (or linear mixed models in JHS to account for family relationships) and performing robust standard error calculations via the 'sandwich' package. These analyses were performed using R version 3.5.3. Because of the small samples within each ethnicity in MESA, we fitted linear models in that study without robust estimation. EWAS results were meta-analyzed across all samples and within each ethnicity using fixed-effect inverse-variance weighted methods implemented in METAL. We required a minimum of two studies for meta-analyses. We used an FDR < 0.05 (Benjamini-Hochberg).

EWAS analyses were subsequently performed in HyperGEN (AA), Generation Scotland (EA), and CATHGEN (AA and EA) using the same statistical protocols as in discovery analyses. For trans-ethnic replication, we combined all the replication samples and used a Bonferroni-adjusted *p*-value cutoff for the 78 tests that were performed (*p* = 6.4E−04). We also considered if the direction of effects between discovery and replication samples was concordant. For ethnic-specific replication, we used EA replication samples for EA or H/L discovery meta-analyses, while for AA replication, we used AA replication samples, a Bonferroni-corrected *p* < 2.1E−03, and consistency in direction of effects. We also attempted to replicate DMPs from a published study [11]. This published study used eGFR instead of DMPs as a predictor in models and therefore the estimates were not comparable to our study.

### In silico annotation using eFORGE and eFORGE-TF, and pathway analyses

We performed functional overlap analysis of DMPs with eFORGE version 2.0, analyzing the default top 1000 probes from EA, AA, H/L, and all-ethnic probe sets for overlap enrichment across DNase I hotspots from the Roadmap Epigenomics Consortium [32, 33]. To ascertain whether the observed enrichment was robust and associated with top probe sets below the EWAS significance threshold, we applied additional eFORGE analyses across the 5 top EA CpG sets (comprising CpGs 1–1000, 1000–2000, 2000–3000, 3000–4000, 4000–5000, ordered by *p*-value), to detect overlap enrichment across DNase I hotspots from the Roadmap Epigenomics Consortium [32, 33]. We thus performed integrative epigenomics analyses on data from the Roadmap Epigenomics consortium [34] using the eFORGE framework (https://eforge.altiusinstitute.org/) [32, 33]. To further understand eFORGE enrichment results, we performed TF motif analysis on the probes underlying eFORGE tissue-specific enrichment signal for kidney. We used the eFORGE-TF module, seeking to uncover the main TF motifs associated with our DNase I hotspot enrichment for top sites [33]. We used PANTHER analysis via the AmiGO framework to uncover pathways associated with the identified TF motifs [35].

### meQTL data in the whole blood

We used publicly available data from the biobank-based integrative omics study (BIOS) QTL database [36, 37] and cis-meQTL data from the FHS (*n* = 4170 participants, 450K DNAm panel, 1000G imputed data, defined within 2-Mb window) [38]. We also used meQTL data from mQTLdb [39].

### meQTL in normal kidney tissue

We analyzed a total of 211 individuals with matching kidney genome-epigenome information from TRANScriptome of renaL humAn TissuE Study (TRANSLATE), an extension of the TRANSLATE study (TRANSLATE-T), Renal gEne expreSsion and PredispOsition to cardiovascular and kidNey Disease (RESPOND), and molecular analysis of mechanisms regulating gene expression in post-ischemic injury to renal allograft (REPAIR) (*n* = 192), in addition to normal samples with available genotype and kidney DNAm profiles from the National Institutes of Health (NIH) Tissue Cancer Genome Atlas (TCGA) (*n* = 19) [40–43]. Kidney tissue samples from TRANSLATE, RESPOND, and TCGA were taken from the healthy, unaffected by cancer part of the organ after elective nephrectomies, and renal specimens in TRANSLATE-T and REPAIR studies were collected as pre-implantation biopsies from deceased donors’ kidneys before transplantation [40, 42, 43]. Information on local recruitment teams, genotyping, and DNAm methods are included in Additional file 2: Supplementary Methods. In total, 374,826 CpG sites were available for further analyses after quality control filters. For post-EWAS DMP analysis, we conducted kidney cis-meQTL analysis on 62 DMPs (out of 374,826) from the study. For GWAS SNP analysis, we conducted kidney cis-meQTL analysis on a set of published eGFR GWAS SNPs [44]. The cis-meQTL analysis was conducted on 195 kidney DNA samples that passed all quality control criteria. For analysis, we used the FastQTL pipeline [45]. We used normalized *M*-values and genotype information for all genotyped and imputed variants passing the quality control filters under an additive mode of inheritance. Regression models included age, sex, genotyping array, source of tissue indicator (nephrectomy/kidney biopsy), the top three PCs derived from genotyped autosomal variants (genotype PCs), and six PCs derived from methylation array control probes (methylation PCs). The FastQTL cis-region was defined as ±1 Mb from each tested CpG/SNP position. For EWAS DMP blood-kidney meQTL comparison analysis, we compared DMP+kidney meQTL CpGs to blood meQTL CpGs from mQTLdb and calculated the percentage of overlap for data from both sources.

### eFORGE analysis for kidney meQTL CpGs

Standard eFORGE analyses were performed using default settings. For kidney meQTL CpGs, we analyzed eGFR SNPs overlapping kidney DNase I hotspots (top category from https://forge2.altiusinstitute.org/files/0x454A546E8E9C11EA8198793F5BFE3F98/index.html). We searched for these SNPs in the kidney meQTL file, obtaining preliminary associated meQTL CpGs (nominal *p*-value < 0.05) and preliminary non-associated meQTL CpGs (nominal *p*-value > 0.95), for the same set of SNPs. Standard eFORGE analyses were performed on both of these sets.

## Results

### Overview of EWAS results

**Fig. 1:**
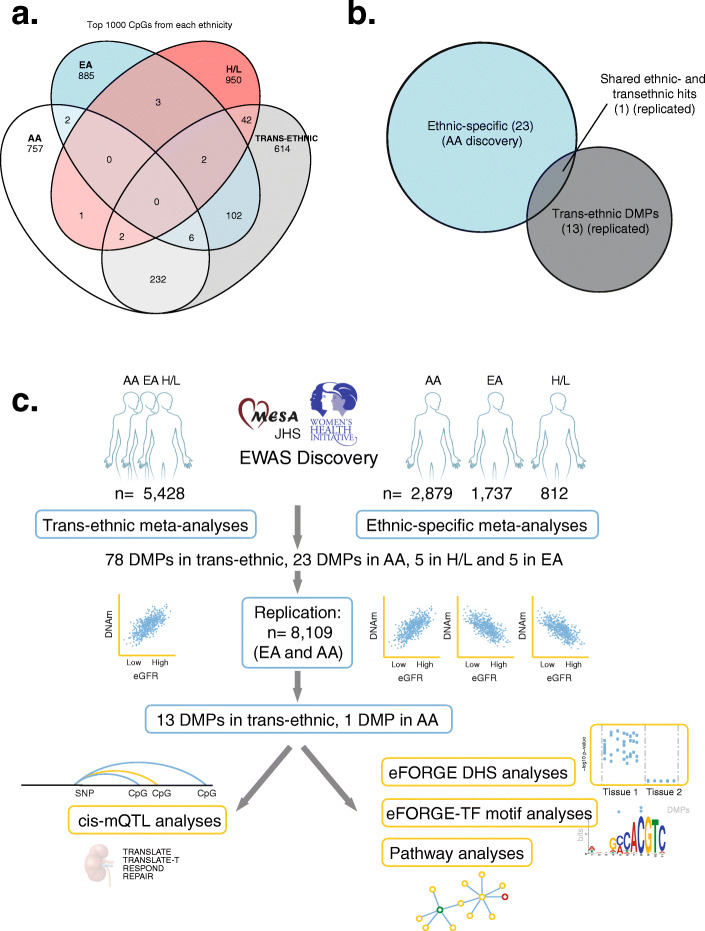
Overview of trans-ethnic and ethnic-specific CpGs associated with kidney function. **a** Venn diagram showing trans-ethnic and unique CpGs across the top 1000 sites for European Americans (EA), African Americans (AA), Hispanic/Latino (H/L), and trans-ethnic groups. **b** Euler diagram showing the number of overlapping CpGs (1) between trans-ethnic replicated DMPs (13) and discovery ethnic-specific DMPs for African Americans -AA- (5). **c** Study design, consortium information, sample size, and the number of significant DMPs for both trans-ethnic and ethnic-specific EWAS analyses. Details shown both for discovery (top) and replication analyses (bottom). For these analyses, consortia include the Women’s Health Initiative (WHI), the Jackson Heart Study (JHS), MESA, HyperGEN, Generation Scotland, and CATHGEN. In addition, we used kidney DNAm data from the TRANSLATE, TRANSLATE-T, RESPOND, and REPAIR studies for *cis*-meQTL analyses and Roadmap Epigenomics data for eFORGE DHS analyses

The study design is shown in Fig. 1 and Additional file 1: Fig. S1. Discovery EWAS meta-analyses included up to 5428 individuals, with 2879 AA, 1737 EA, and 812 H/L participants (Additional file 3: Table S1). Quality control of DNAm data and protocol analyses for each study are shown in Additional file 3: Table S2. We performed analyses within each study and ethnic group using standard statistical protocols followed by meta-analyses of the overall samples and within each ethnicity. The quantile-quantile and Manhattan plots for meta-analyses are shown in Additional file 1: Fig. S2. Lambdas for meta-analyses were 0.987 (AA), 1.001 (EA), 1.065 (H/L), and 1.194 (trans-ethnic meta-analysis). Across our discovery analyses, we identified a total of 93 DMPs associated with eGFR at an FDR of 0.05. Of these, 78 DMPs were identified in trans-ethnic meta-analyses, 23 DMPs in the meta-analysis of AA, 5 in the meta-analysis of H/L, and 5 in the meta-analysis of EA, with some overlap in DMP findings between trans-ethnic and ethnic-specific results.

Indeed, most of the DMPs identified in trans-ethnic analyses were also present in one or more ethnic groups (Fig. 1a). However, the overlap between DMPs of AA and EA was small. Trans-ethnic replication included up to 8109 participants from three studies (Generation Scotland, CATHGEN, and HyperGEN), composed of participants of EA and AA (9% of replication samples) (Additional file 1: Fig. S1) [11]. Replication of EA or H/L findings included Generation Scotland and CATHGEN EA samples (*n* = 7349) and AA included CATHGEN and HyperGEN samples (*n* = 760). Among the significantly identified trans-ethnic DMPs (Bonferroni corrected *p*-value and consistent direction of effects), 13 of 78 replicated (Table 1), and among the significantly identified DMPs in ethnic-specific meta-analyses, 1 of 5 AA DMPs replicated (cg14871770 at *CYP2C9*) (Additional file 3: Table S3). Despite independent replication in AA, cg14871770 overlapped with trans-ethnic replicated DMPs (Fig. 1b). Twelve additional DMPs had replication (with consistent direction of effects) in trans-ethnic meta-analyses (Additional file 3: Table S3a). The DMP cg14871770 overlapped between trans-ethnic and AA meta-analyses, and the cg17944885 at the *ZNF20-ZNF788P* locus was previously described [11]. Several of the replicated DMPs were expression quantitative trait methylation loci (eQTM) or cis-meQTL CpGs in whole blood in FHS and (BIOS QTL) (Table 1) [36–38]. Replication results for all DMPs are shown in Additional file 3: Tables S3a (trans-ethnic analyses) and Additional file 3: Table S3b (ethnic-specific analyses). Forest plots for each of the replicated DMPs are shown in Additional file 1: Fig. S3. We also replicated 6 DMPs identified in a prior publication (DMPs located at genes *DAZAP1*, *KIAA1549L*, *TUBGCP4/ZSCAN29*, *JAZF1*, *ZNF20-ZNF788P*, *LDB2*) (Additional file 3: Table S4) [11].

**Table 1:** Main findings from trans-ethnic EWAS meta-analyses for 13 replicated DMPs

				Our study discovery		Sample size by DNAm array		Replication GS/CATHGEN/HyperGEN		Combined discovery and replication	
DMP	Chr	Position (hg38)	Gene	Effect	***p***	450K	EPIC 850 K	Effect	***p***	Effect	***p***
cg13235761^	1	203,592,452		− 37.61	4.33E−06	3048	2378	− 25.28	5.08E−05	− 29.81	1.88E−09
cg26099045^*	2	64,064,666		14.82	5.74E−06	3050	2378	11.24	1.01E−04	12.81	3.26E−09
cg04428662^Ω*	4	2,932,461	*MFSD10*	− 30.84	3.21E−06	3049	2378	− 34.94	3.43E−07	− 32.82	5.51E−12
cg23174201^*	5	151,674,695	*SPARC*	− 35.83	5.10E−06	3050	2378	− 34.87	1.72E−07	− 35.27	4.02E−12
cg17170437	6	44,229,461	*SLC29A1*	− 47.62	1.96E−06	N/A	2378	− 18.62	1.49E−04	− 24.24	3.80E−08
cg14871770	10	96,658,622	*CYP2C9*, *CYPC19*	− 53.12	1.98E−06	N/A	2378	− 25.87	1.70E−04	− 33.36	1.23E−08
cg02157636	11	68,709,367	*TESMIN*	− 53.21	2.15E−06	N/A	2378	− 27.25	1.14E−05	− 33.33	8.58E−10
cg26039141^Ω	11	75,402,116	*RPS3*	− 39.14	3.40E−06	3050	2378	− 33.42	1.78E−05	− 36.06	2.90E−10
cg22593432^*	13	32,001,768		− 29.91	1.54E−07	3050	2378	− 17.66	1.79E−04	− 22.64	4.61E−10
cg11789371^*	14	102,085,048	*HSP90AA1*	− 35.40	4.44E−06	3050	2378	− 22.66	3.58E−04	− 27.80	1.41E−08
cg05796561	18	57,128,273		− 45.24	2.96E−06	N/A	2378	− 22.91	1.69E−04	− 29.24	1.41E−08
cg17944885^Ω*	19	12,114,920	*ZNF20, ZNF788P*	− 32.71	1.41E−09	3048	2378	− 16.34	5.67E−07	− 20.72	1.24E−13
cg15787712^	19	13,837,429	*LOC284454*, *MIR23*	− 37.60	6.00E−09	3050	2378	− 28.65	2.06E−05	− 33.30	9.01E−13

*p*-value for replication < 6.4E−04 (Bonferroni correction for 78 DMPs tested). Four of the 13 DMPs that replicated were non-450K probes (cg17170437, cg14871770, cg02157636, cg05796561). Results from combined discovery and replication are also shown

*GS* Generation Scotland, *N/A* not applicable

^*cis*-meQTL from the BIOS QTL [36, 37]

ΩExpression quantitative trait methylation (eQTM) data from the BIOS QTL (enrichment *p*-value <0.01) [36, 37]

*cis-meQTL in the Framingham Heart Study [38]

### Overlap of eGFR-associated DMPs with genes and regulatory elements

**Fig. 2:**
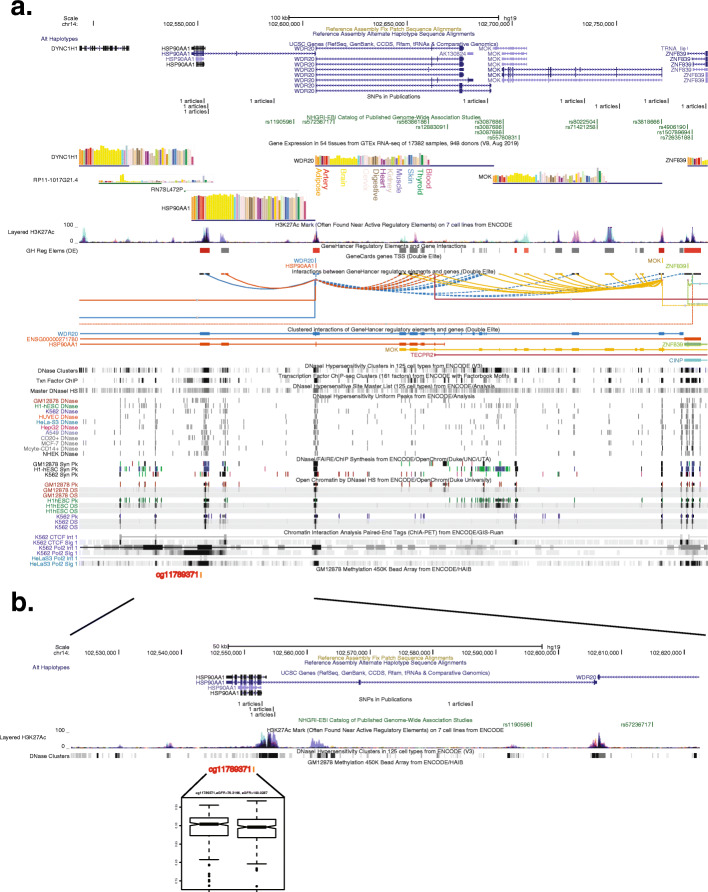
eGFR-associated differentially methylated position cg11789371. **a** *HSP90AA1* gene browser shot showing (from top to bottom) genome coordinates, local genes, NHGRI/EBI GWAS catalog SNPs, GTEx gene expression quantified via RNA-seq across different tissues, H3K27ac peaks across 7 ENCODE cell lines, GeneHancer regulatory elements, Genecards TSSs, GeneHancer chromatin interactions, ENCODE chromatin accessibility and chromatin interaction tracks, and location for eGFR-associated DMP cg11789371. **b** Expanded browser shot showing genome coordinates, local genes, NHGRI/EBI GWAS catalog SNPs, H3K27ac peaks across 7 ENCODE cell lines, and a boxplot indicating DNAm values at cg11789371 for bottom and top quartiles of eGFR, respectively. These data indicate our DMP overlaps an intron of *HSP90AA1*, a gene expressed in kidney tissue, and a DHS from ENCODE, which was detected in kidney tissue. Our DMP is also proximal to an H3K27ac peak, an RNA Polymerase 2 region determined by ENCODE ChIA-PET across several cell lines, and the promoter of *HSP90AA1*. All browser shots were generated using the UCSC genome browser (https://genome.ucsc.edu/) on human genome build hg19

To understand the regulatory context of our DMPs, we annotated the 13 replicated significant DMPs with the closest gene and other information including epigenomic peaks, tissue-specific gene expression via RNA-seq, and chromatin interaction annotations. One of our DMPs (cg11789371) is located in an intron of *HSP90AA1*, a gene involved in protecting kidney tissue from inflammation, ischemia, and oxidative damage, and assisting with cellular repair (Fig. 2) [46]. HSP90 (heat shock protein 90) has a physiological role in eGFR regulation through the nitric oxide pathway and is a drug target candidate for kidney diseases [46]. Indeed, inhibition of HSP90 has been shown to reduce eGFR in animal models [46]. DMP cg11789371 overlaps epigenomic annotations from the ENCODE consortium including DNase I hypersensitive sites in kidney cells, among other cell types. This DMP is a cis-meQTL CpG regulated by rs11621083, a variant located upstream in an intron of *HSP90AA1*. The DMP also forms part of a GeneHancer interacting site, contacting the promoter of *WDR20* and an alternative promoter of *HSP90AA1* which is located 50 kb away. The entire region surrounding this DMP contains a number of genes expressed in the kidney, several of which seem to interact with these two promoters. Genes from this locus presenting RNA-seq expression in the kidney include *DYNC1H1*, *MOK*, *ZNF839*, *WDR20*, and *HSP90AA1*. Taken together, annotations for our DMP cg11789371, which overlaps an intron of *HSP90AA1*, a gene associated with eGFR regulation, suggest a potential link between DNA methylation and eGFR regulation through the nitric oxide pathway.

### DMPs for eGFR are enriched for kidney regulatory function related to kidney development

**Fig. 3:**
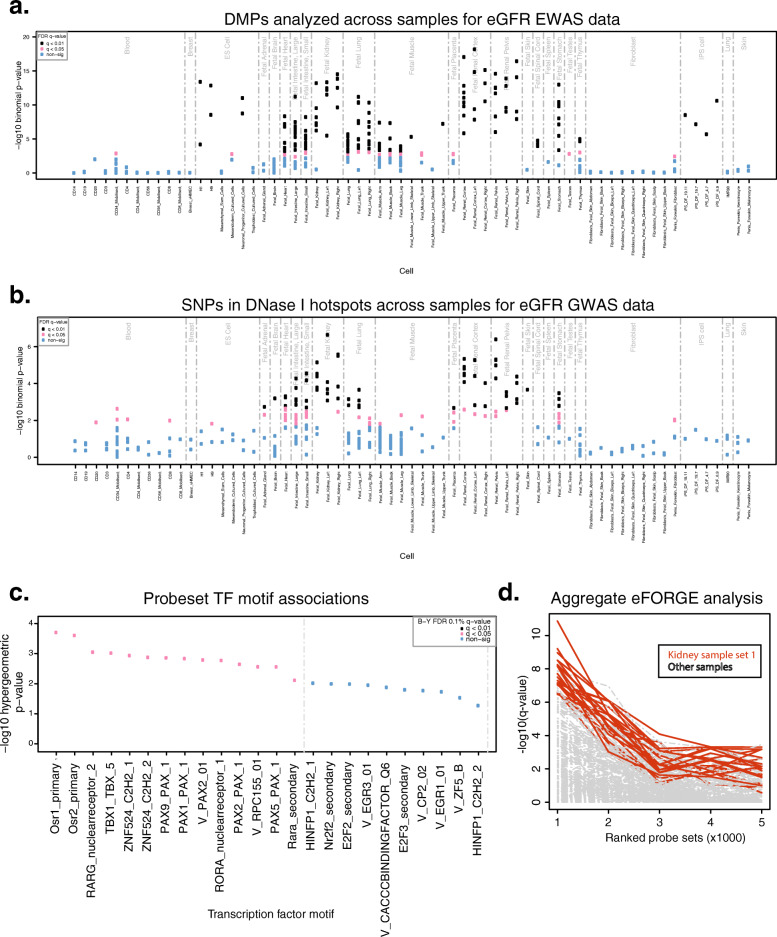
Tissue-specific integrative analysis indicates potential effect on kidney and relation with eGFR GWAS loci. **a** eFORGE analysis for top 1000 eGFR CpGs: the *x* axis indicates tissues/cell type samples used in the analysis; the *y* axis shows eFORGE enrichment (−log10 *p*-value) of the CpG set with DNase I hotspots for a range of tissue samples (significant samples in black). The highest ranked sample set (highest black points) shows the most significant enrichment is for kidney samples, which are highly ranked for the top 1000 CpGs associated with eGFR. **b** FORGE2 analysis for eGFR SNPs from GWAS catalog: the *x* axis indicates tissues/cell type samples used in the analysis; the *y* axis shows FORGE2 enrichment (−log10 *p*-value) of the SNP set with DNase I hotspots for a range of tissue samples (significant samples in black). The highest ranked sample set (highest black points) shows the most significant enrichment also is for kidney samples, which are highly ranked for the top 249 SNPs associated with eGFR (taken from the GWAS catalog, https://www.ebi.ac.uk/gwas/, downloaded 10 April 2020). **c** TF motif enrichment results for EA probes driving eFORGE tissue-specific enrichment signal: the *x* axis indicates TF motifs from TRANSFAC, JASPAR, Taipale/SELEX, and Uniprobe databases; the *y* axis shows eFORGE-TF enrichment (−log10 hypergeometric *p*-value) of the input DMP set with TF motifs overlapping open chromatin sites for fetal kidney samples. Enrichment values for each TF motif are colored according to BY FDR-corrected *q*-value. A number of TF motifs involved in kidney development overlap top EA probes including *OSR1*, *OSR2*, *TBX1*, and *PAX2*. **d** Aggregated eFORGE results for EA probes: the *x* axis indicates sets of the top ranked DMPs used in the analysis (each set contains 1000 DMPs); the *y* axis shows eFORGE enrichment (−log10 *p*-value) of each of the DMP sets with open chromatin sites for kidney (red) and other tissue samples (gray). The highest ranked probe set (set 1, left) shows the most significant enrichment for kidney samples, which remain highly ranked for probe sets 2–5, in decreasing order of study *p*-value

To further understand the regulatory potential and chromatin context of our EWAS findings across different tissues, we performed integrative epigenomics analyses on data from the Roadmap Epigenomics consortium [34] using the eFORGE framework (https://eforge.altiusinstitute.org/) [32, 33]. We found enrichment for kidney-specific DNase I hotspots, which has also been described in GWAS of eGFR (Fig. 3a, d) [4, 47]. eFORGE showed consistent enrichment results for kidney-specific DNase I hotspots when applied to the top EA discovery probes, showing a corresponding trend with study *p*-value (analyses performed using the “EPIC” setting with 1000 repetitions, BY correction). In addition, analysis with eFORGE-TF uncovered significant enrichment for several transcription factor (TF) motifs (including motifs for *OSR1*, *OSR2*, *TBX1*, and *PAX2*). *OSR1*, *OSR2*, *TBX1*, and *PAX2* have all been shown to have roles in kidney development (Fig. 3c) [48–50]. To further understand the pathways underlying TF motif enrichment, we performed PANTHER pathway analysis using significant TF motifs [35]. This analysis uncovered pathways associated with kidney development, including metanephros development (8.1 × 10^−3^), mesonephros development (9.7 × 10^−3^), and retinoic acid receptor signaling pathway (6.4 × 10^−3^) (Additional file 3: Table S5). Overall, these findings suggest that epigenetic changes related to eGFR are enriched in kidney regulatory regions and pathways related to kidney development.

### eGFR GWAS variants, meQTL CpGs, and tissue-specific DNase I hotspots

**Fig. 4:**
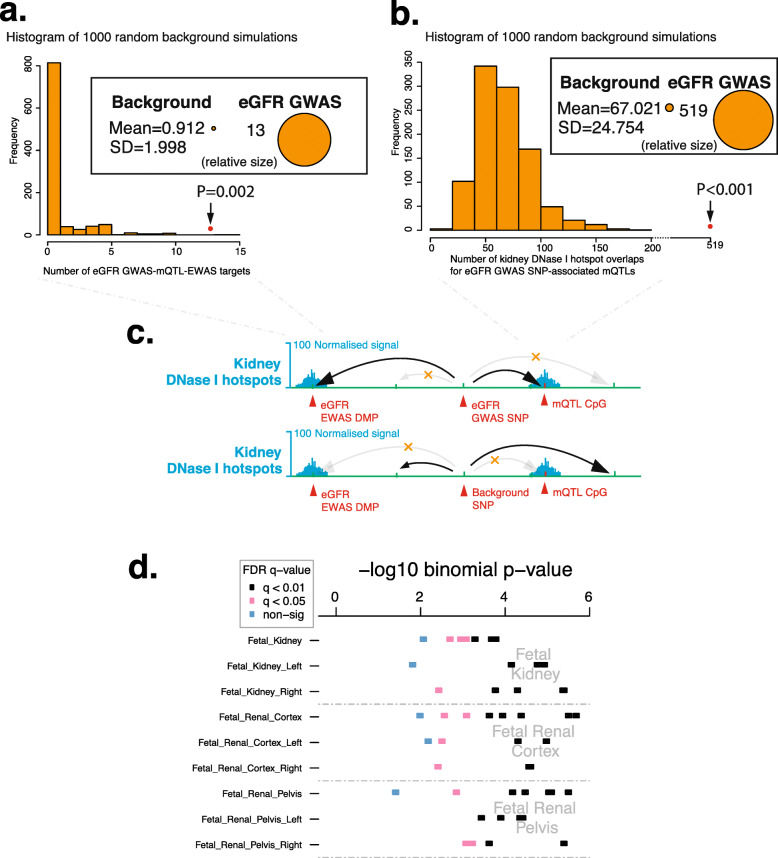
eGFR EWAS CpGs present a significant overlap with eGFR GWAS-driven meQTL effects. **a** Histogram of 1000 random background simulations (249 random SNPs each), for EWAS-meQTL overlap across the ARIES blood meQTL dataset (http://www.mqtldb.org/). Two hundred forty-nine unique significant SNPs from the eGFR GWAS by Hellwege et al. yield 13 SNP-meQTL-EWAS DMP sites in the Aries cohort (*p* = 2.0E−03, empirical test, red dot and arrow), while background SNP sets overlap a mean of 0.912 SNP-meQTL-EWAS sites. **b** Histogram of 1000 random background simulations (249 random SNPs each), for meQTL-kidney DNase I hotspot overlap across Roadmap Epigenomics “Kidney” sample datasets (https://egg2.wustl.edu/roadmap/web_portal/). Two thousand seven hundred thirty-three meQTL targets of 249 unique significant SNPs from the eGFR GWAS by Hellwege et al. overlap Roadmap kidney DNase I hotspots 519 times (*p* < 0.001, empirical test, red dot and arrow), while background SNP sets overlap Roadmap kidney DNase I hotspots a mean of 67.021 times (SD = 24.754). **c** Schematic showing the association of eGFR GWAS SNPs with meQTL target CpGs and eGFR EWAS CpGs (both in red text), some of which overlap kidney-specific DNase I hotspots (shown in blue, arrows indicate statistical association—not genomic contact). For comparison, a representation of a background SNP is shown. **d** Results from eFORGE analysis of significant ARIES meQTL CpGs associated with eGFR GWAS SNPs, indicating a higher-than expected overlap with the kidney, renal cortex, and renal pelvis DNase-seq hotspots (for additional results, see Additional file 1: Fig. S5)

Previous reports and our analyses confirm that eGFR GWAS variants are enriched for kidney DNase I hotspots (Fig. 3b, Additional file 1: Fig. S4) [4, 47], indicating that genotypes might function through kidney-specific regulatory pathways, potentially converging with identified EWAS DMPs through mechanisms which are not fully understood. To explore this further, we used mQTLdb [39] to identify meQTL CpG targets of eGFR-associated GWAS SNPs obtained from the GWAS catalog [44, 51]. These significant meQTL CpGs linked to GWAS SNPs revealed significant overlap with eGFR EWAS sites in our study (*p* < 0.002, Fig. 4a) and presented significant enrichment for kidney, renal cortex, and renal pelvis, among other tissues (Fig. 4b, d, Additional file 1: Fig. S5). These results support a model in which meQTL CpGs linked to eGFR GWAS SNPs overlap with EWAS DMPs and tend to localize to kidney DNase I hotspots, with potential involvement in their regulatory action (Fig. 4c). To assess these findings in human kidney tissue, we analyzed meQTL CpGs of eGFR SNPs localizing to kidney DNase I hotspots. The meQTL CpGs were identified from DNAm data from 195 normal kidney tissue samples (acquired from elective nephrectomies—taking the non-cancer affected segment—or from kidney donors) as reported previously [40–42]. While kidney meQTL CpGs not associated with eGFR SNPs (nominal *p*-value> 0.95) showed no enrichment in kidney DNase I hotspots (Additional file 1: Fig. S6), the kidney meQTL CpGs with a nominal *p*-value < 0.05 were enriched in kidney DNase I hotspots (Additional file 1: Fig. S7) for the same GWAS SNPs. These results support the aforementioned findings using eGFR SNP-associated meQTL CpGs from mQTLdb [39]. Additionally, we evaluated the overlap between kidney meQTL CpGs and blood meQTL CpGs (mQTLdb) for our top EWAS DMPs. The majority (58.3%) of these kidney meQTL CpGs were also mQTL CpGs in blood.

## Discussion

### Main findings

This study used trans-ethnic and ethnic-specific analyses of multi-ethnic cohorts to identify and replicate DMPs associated with eGFR. Our main findings include replicated DMPs at 13 sites from trans-ethnic analyses and 1 DMP from AA-specific analyses, which may reflect the larger AA discovery sample compared to other ethnic groups. All associations were newly identified in this study, except for the *ZNF20-ZNF788P* locus, which was previously described in two separate eGFR EWAS, including a population-based cohort and an HIV-infected cohort [11, 52]. Several DMPs were associated with accessible chromatin sites in kidney tissue, suggesting a regulatory role for these sites. Overall, our study identified 12 previously unreported DMPs that replicated, in addition to 6 previously published DMPs for eGFR among our 93 discovery DMPs [11]. These findings support an association between DNAm and eGFR, reflecting a convergence of lifetime influences of genetic effects, lifestyle, behaviors, and environmental exposures [53–55].

### Differences between ethnicities

Our approach of studying multi-ethnic groups identified DMPs from trans-ethnic and ethnic-specific analyses. This approach contrasts with a prior study that performed separate EA and AA analyses with cross-replication across these groups [11]. Across our two largest ethnic-specific samples (AA and EA), there was little overlap between DMPs from discovery analysis, although several discovery ethnic-identified DMPs did overlap with trans-ethnic findings (Fig. 1a). The DMP cg14871770, which is located between *CYP2C9* and *CYP2C19* (cytochrome P450 family 2 subfamily C members 9 and 19), was identified both in ethnic-specific and trans-ethnic analysis. The closest GWAS SNP to this DMP is rs4110517, a SNP associated with blood pressure identified in a multi-ethnic cohort [56]. *CYP2C9* and *CYP2C19* encode members of the cytochrome P450 superfamily of enzymes. Cytochrome P450 proteins are monooxygenases that catalyze many reactions involved in the synthesis of cholesterol, other steroids, and other lipids, and in drug metabolism. *CYP2C19* genotype is associated with the metabolism of compounds influencing both renal function and hypertension [57]. The relevance of ethnic-specific findings to clinical phenotypes will need further evaluation in studies with larger samples inclusive of multiple ethnicities to define improved DNAm signatures for eGFR that may be unique to one single ancestry. Our findings suggest reduced utility of DNAm biomarkers for eGFR in diverse populations if discovery EWAS is performed in a single homogenous population.

### Relevance of *HSP90AA1* locus

Among other findings, we report a replicated trans-ethnic DMP (cg11789371) associated with eGFR that localizes to an intron of *HSP90AA1* (heat shock protein 90 alpha family class A member 1), a gene expressed in podocytes, parietal epithelial cells, proximal tubular cells, endothelium, and mesangial cells in normal kidney tissue, with gene expression increasing in glomerulonephritis and acute kidney injury [46]. The gene product, HSP90, regulates renal blood flow and eGFR through nitric oxide metabolism and plays a role in protein folding [46]. HSP90 and other heat shock proteins are candidate drug targets for a variety of kidney diseases [46, 58]. Treatment with radicicol, an inhibitor of HSP90, has been shown to reduce eGFR in animal models [58]. DMP cg11789371 overlaps an accessible chromatin region in kidney cells, and a GeneHancer interacting site contacting the promoters of *WDR20* and *HSP90AA1*. These and other different annotations point to a potential regulatory role in kidney tissue for this eGFR-associated DMP.

### Integrative epigenomics and pathway analysis

We detected a significant overlap of eGFR GWAS SNP-associated meQTL CpGs with kidney DNase I hotspots. Additionally, our eGFR EWAS DMPs were enriched for kidney DNase I hotspots. These findings suggest potential links between the regulatory action of both genotypes and epigenetic DNAm elements. Importantly, these integrative epigenomic analyses considered all tissues available instead of only kidney tissue.

While some regions of the methylome show tissue specificity [59], EWAS have also shown that some DMPs are shared across different tissues, e.g., the *AHRR* locus DNAm patterns in response to smoking are shared across multiple tissues [60]. Pan-tissue findings for *AHRR* suggest similar underlying pathways in response to the same environmental stimulus [60]. Regarding discrepancies in trans-ethnic results, both genetic and environmental differences could be at play, potentially interacting with each other. In this context, our findings of both a kidney-specific DNase I hotspot and GWAS meQTL enrichment for a whole blood-based EWAS could be due to both genetic and environmental origins and warrant further research of kidney tissue DNAm in association with eGFR. Indeed, such results raise the intriguing hypothesis that tissue-specific enrichments observed separately in GWAS and EWAS might be related by the same genomic variants, thus aiding the integration of both approaches (Fig. 4a). It is important to highlight that both whole blood and kidney tissue eQTLs were obtained from individuals of European ethnicity [40–43].

The identification of eGFR DNAm signature-associated pathways is an important step towards characterizing epigenetic mechanisms for this physiologic trait and may provide clues to underlying mechanisms for CKD. Identified DMPs highlight an association with pathways of kidney development, which can influence nephron endowment at birth and subsequent CKD risk [48–50, 61]. Our in silico results were influenced by our DNAm findings in healthy adult kidney. Therefore, pathway results from this study support epigenetic effects during developmental windows with long-term influence on eGFR, which warrant further investigation.

### Limitations

This study is limited by the use of whole blood as the main tissue (chosen due to its availability). The discovery datasets included both the Illumina 450K and the EPIC 850K arrays, which contributed to differences in sample sizes and power to detect associations for some CpGs (Table 1). Post hoc power analyses suggest adequate power to detect DNAm differences of the range observed in the study (Additional file 2: Supplementary Methods). The methods for normalization of the DNAm beta values and study-specific quality control varied (Additional file 3: Table S2). However, we applied standardized protocols for data harmonization and statistical analyses in addition to stringent quality control as part of our meta-analyses. Our findings showed no heterogeneity of effects across studies (Additional file 1: Fig. S3), suggesting that results are robust to study-specific quality control and normalization procedures. Additionally, our reported DMPs replicated in independent samples, further validating our results. It is important to consider whether these DNAm sites associated with eGFR in blood are also applicable to effects in kidney tissue. We attempted to answer this by examining our identified meQTLs in normal kidney tissue in the TRANSLATE study. However, kidney tissue studies still have small sample sizes and lack ethnic diversity. While epigenomic mapping consortia such as Roadmap Epigenomics and ENCODE have made important steps to increase the free availability of a wide range of tissue and cell type-specific datasets, the important issue of including additional epigenomics mapping data sets for other ancestries remains to be addressed. It is important to highlight that we only observe eFORGE kidney enrichment for top EA probes. While this kidney-specific DNase I hotspot enrichment has been further confirmed by analyzing ranked DMPs in study *p*-value order (Fig. 3d), it is not apparent for DMPs from other ethnicities, or for DMPs for analysis comprising all ethnicities. DNase-seq datasets for these samples originate from the Roadmap Epigenomics consortium [34], which focused mainly on tissue samples obtained from EA individuals. Without datasets from diverse ethnic groups, it will be difficult to conclusively study inter-ethnic epigenomic variability or perform tissue-specific analyses for loci from GWAS and EWAS performed on individuals of non-European origin.

## Conclusions

We identified trans-ethnic and ethnic-specific differential DNAm positions, validated prior published associations, and showed that several eGFR DMPs identified in this study replicated in independent samples. We have also shown that some of the DMPs are meQTL CpGs, many of which are associated with pathways relevant for kidney tissue regulation and development. Our findings include a DMP at *HSP90AA1*, a gene involved in the regulation of eGFR in kidney tissue. Identification of trans-ethnic and ethnic-specific DMPs and elucidation of their potential functional impact are preliminary steps towards identifying disease-associated epigenetic mechanisms that are specific to a particular population or shared across different populations.

## Supplementary Information

**Additional file 1: Supplementary Figures**. **Figure S1 to S7**. Study design, QQ plots, Manhattan plots, forest plots and eFORGE/FORGE2 analyses.

**Additional file 2: Supplementary Methods**. Description of different constituent cohorts and studies, in addition to power analyses.

**Additional file 3: Supplementary Tables**. **Tables S1 to S5**. Descriptive characteristics, quality control and processing, and results.

## Data Availability

Investigators interested in retrieving the controlled-access data at dbGap for WHI-BAA23 and MESA should apply using identifiers phs000200.v10.p3 and phs001416.v1.p, respectively (available online at https://www.ncbi.nlm.nih.gov/projects/gap/cgi-bin/study.cgi?study_id=phs000200.v10.p3 and https://www.ncbi.nlm.nih.gov/projects/gap/cgi-bin/study.cgi?study_id=phs001416.v1.p1). The summary results from this study are placed in dbGaP with accession number phs000930.v8.p1. While awaiting data release via dbGaP, data are available at https://sph.unc.edu/wp-content/uploads/sites/112/2021/02/EWAS_COGENT.tar. Access to the WHI-EMPC DNA methylation dataset is available upon request to www.whi.org. DNA methylation datasets from JHS and HyperGEN have been recently generated and upload to dbGap is ongoing. JHS data are available on request from www.jacksonheartstudy.org and HyperGEN data are available from the corresponding author from Ammous et al. [62]. Currently, JHS and WHI datasets are available through a scientific review application process directed to each respective study publication and presentation committee. Data can be obtained from the coordinating center of WHI and JHS after signing a data use agreement with the study. For research projects that meet the rules for access, CATHGEN data are available from the CATHGEN Steering Committee. Relevant requests are to be sent to the contact person at the CATHGEN Steering Committee, Melissa Hurdle (melissa.hurdle@duke.edu). According to the terms of consent for GS participants, access to individual-level data (omics and phenotypes) must be reviewed by the GS Access Committee. Applications should be made to access@generationscotland.org. The source code used for the eFORGE analyses is publicly available at https://github.com/charlesbreeze/eFORGE [32] and https://github.com/charlesbreeze/eFORGE-TF [33].
