# Supplementary material for: Epigenome-wide association study of kidney function identifies trans-ethnic and ethnic-specific loci
Source: Genome Med. 2021 Apr 30;13:74. doi: 10.1186/s13073-021-00877-z (PMC8088054; doi:10.1186/s13073-021-00877-z)
Supplement: Supplementary file 1 — Additional file 1: Supplementary Figures. Figure S1 to S7. Study design, QQ plots, Manhattan plots, forest plots and eFORGE/FORGE2 analyses. [file 13073_2021_877_MOESM1_ESM.pdf]

Supplementary Figures

Epigenome-wide association study of kidney function identifies shared and ethnic-specific loci

Study design, discovery and replication analyses

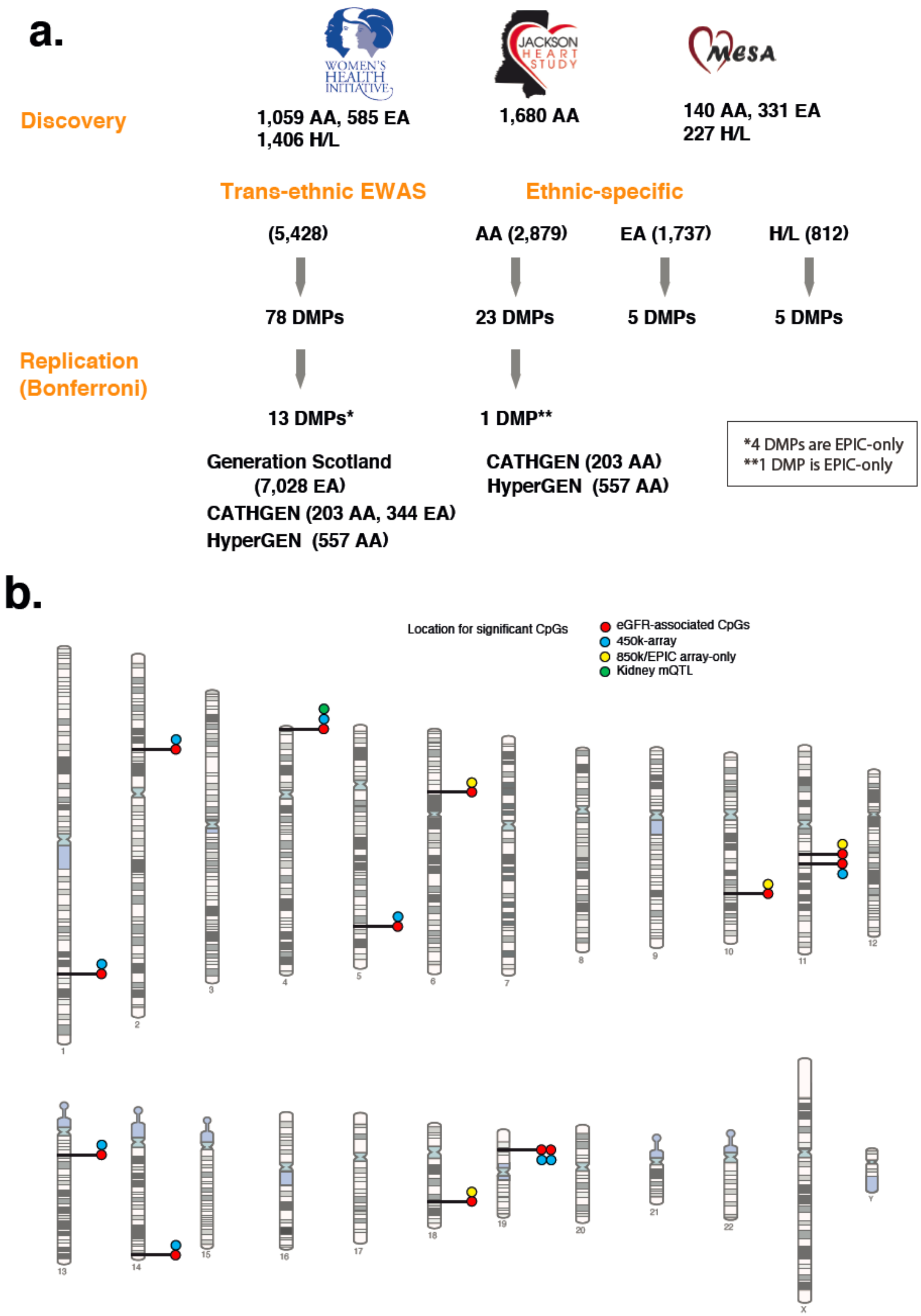

Figure S1: Study design details and main findings: (a) consortium information, sample size and number of significant DMPs for both trans-ethnic and ethnic-specific EWAS analyses. Details shown both for discovery (top) and replication analyses (bottom). Studies/cohorts include the Women's Health Initiative (WHI), the Jackson Heart Study (JHS), the MESA cohort, the Generation Scotland cohort, and CATHGEN and HyperGEN studies, among others. (b) Karyotype showing the genomic location of the top 13 trans-ethnic significant CpGs, along with annotations for 450k/EPIC array status and mQTL status in kidney (determined via lookup of kidney mQTL CpG sites from REPAIR, RESPOND, TRANSLATE-T and TRANSLATE studies).

a. Trans-ethnic meta-analysis QQ plot (left) and Manhattan plot (right)

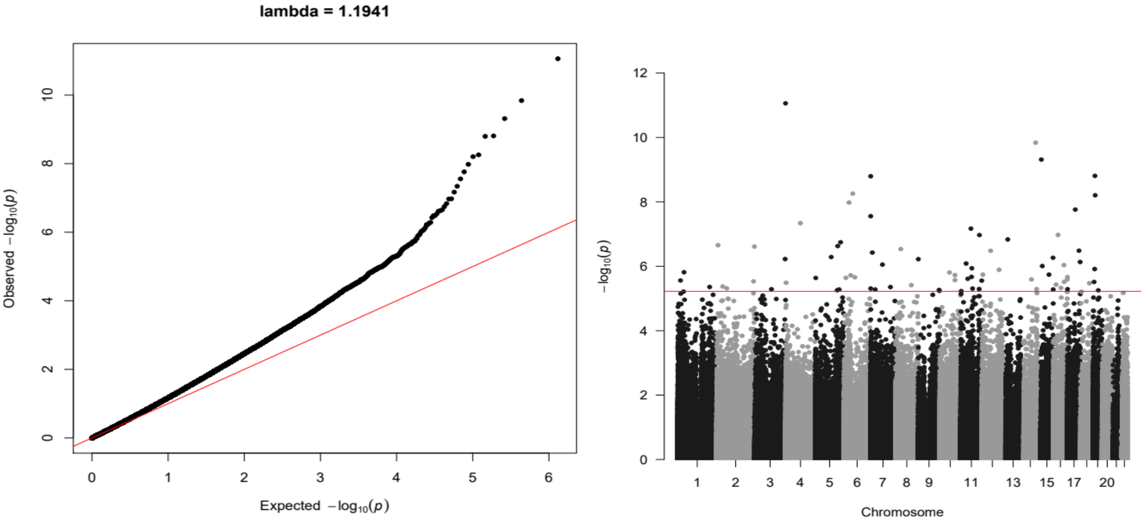

b. African American meta-analysis QQ plot (left) and Manhattan plot (right)

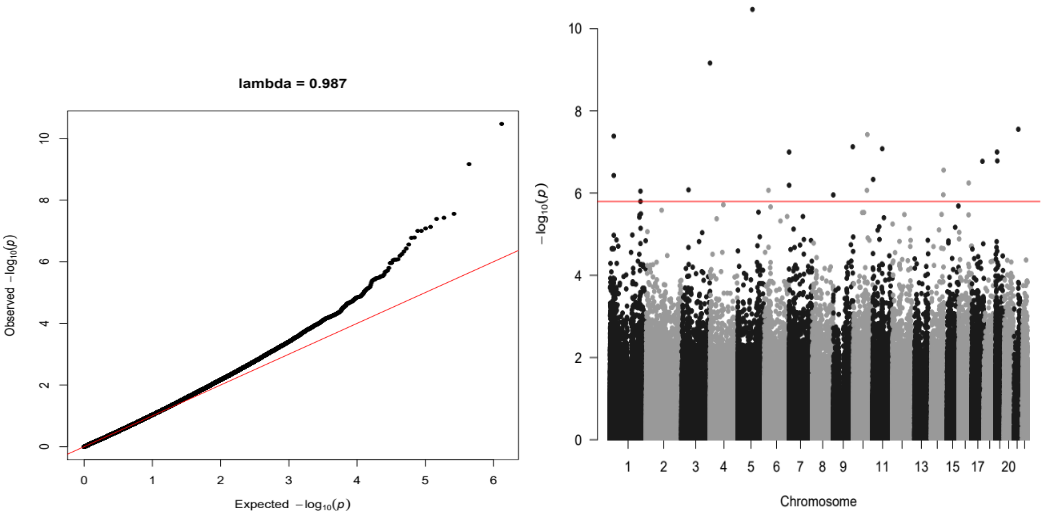

c. European American meta-analysis QQ plot (left) and Manhattan plot (right)

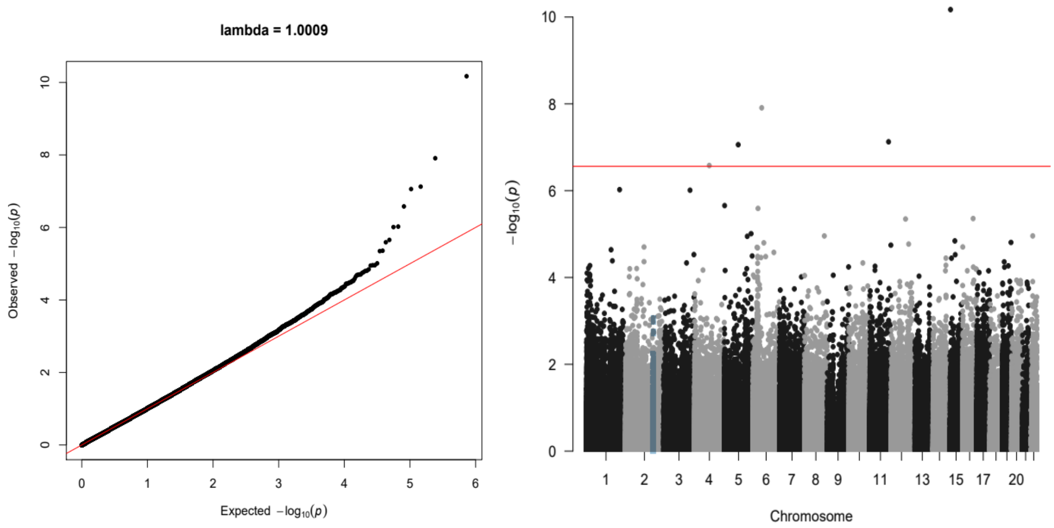

d. Hispanic American meta-analysis QQ plot (left) and Manhattan plot (right)

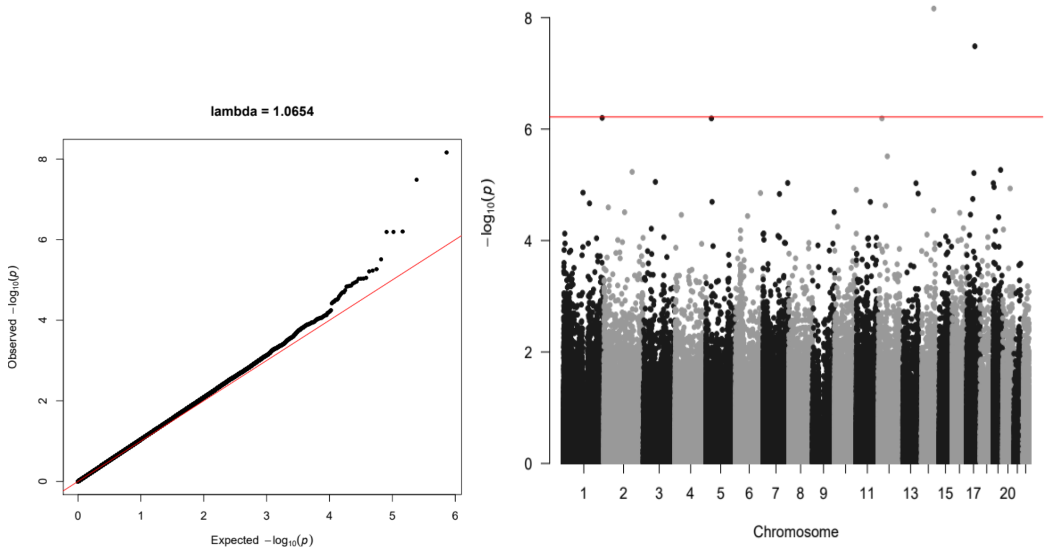

Figure S2. **(a-d):** Quantile-quantile (QQ) and Manhattan plots for trans-ethnic and ethnic-specific meta-analyses. QQ plots are shown on the left and Manhattan plots are shown on the right of each panel.

a. Forest plot for probe cg13235761

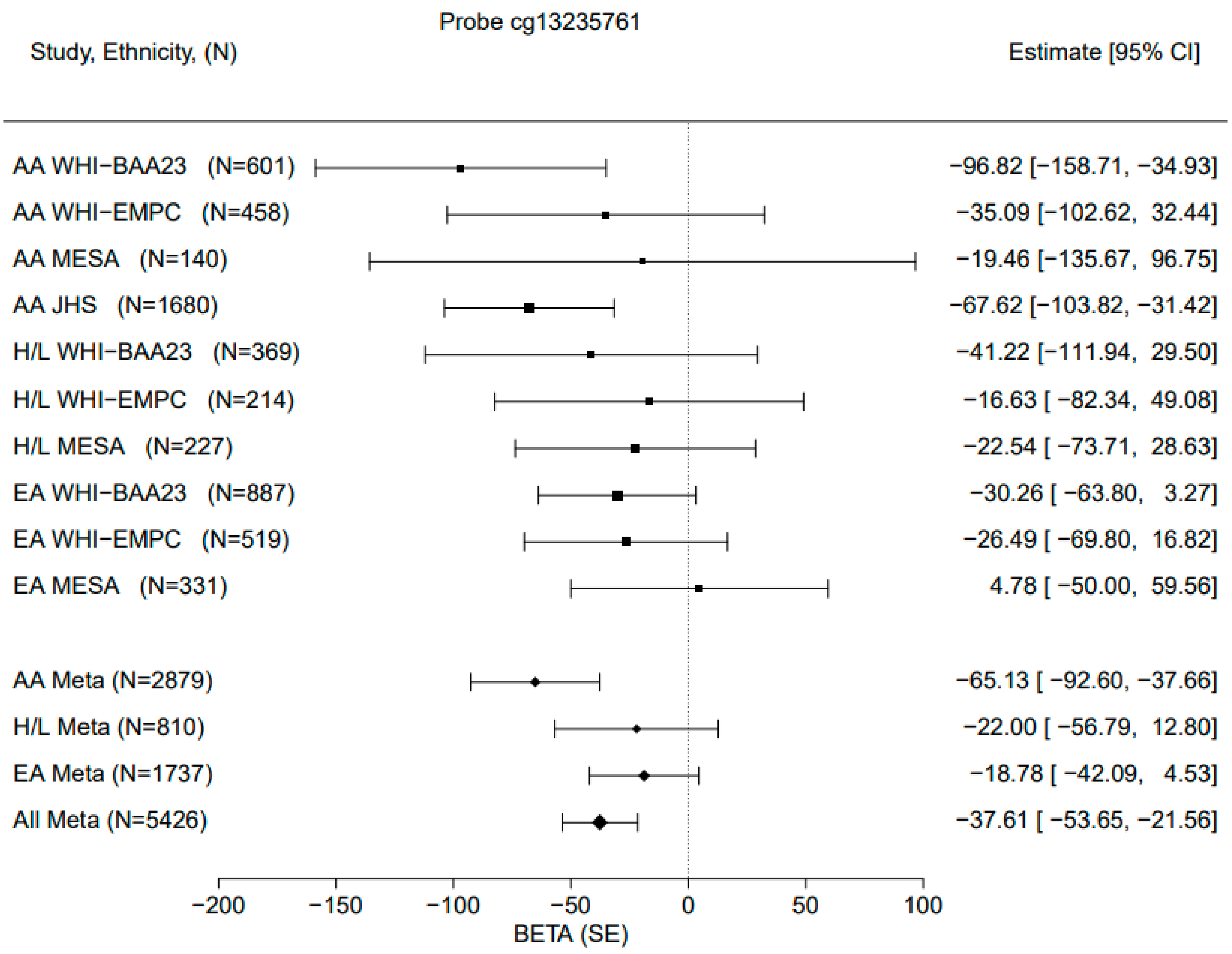

b. **Forest plot for probe cg26099045**

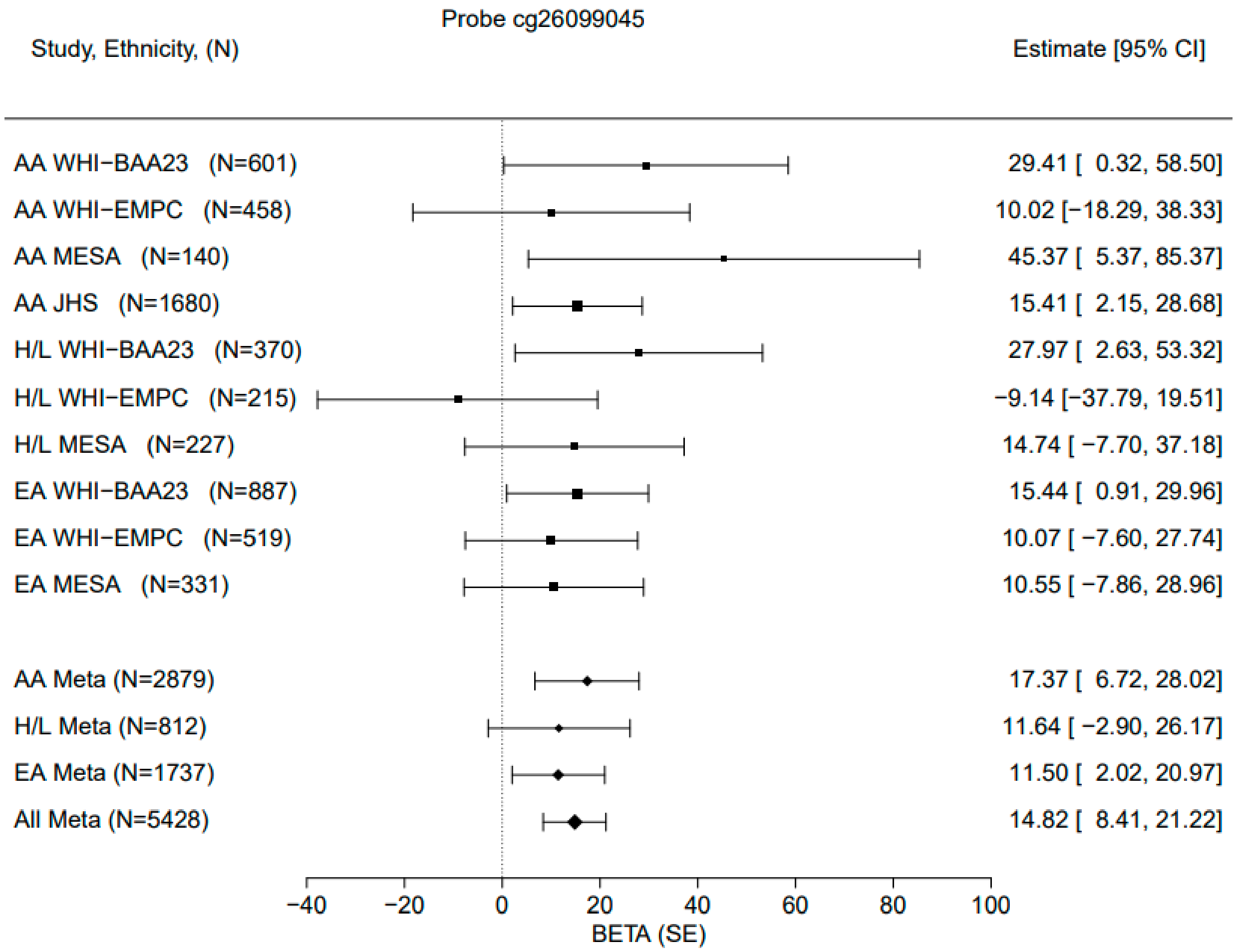

c. **Forest plot for probe cg04428662**

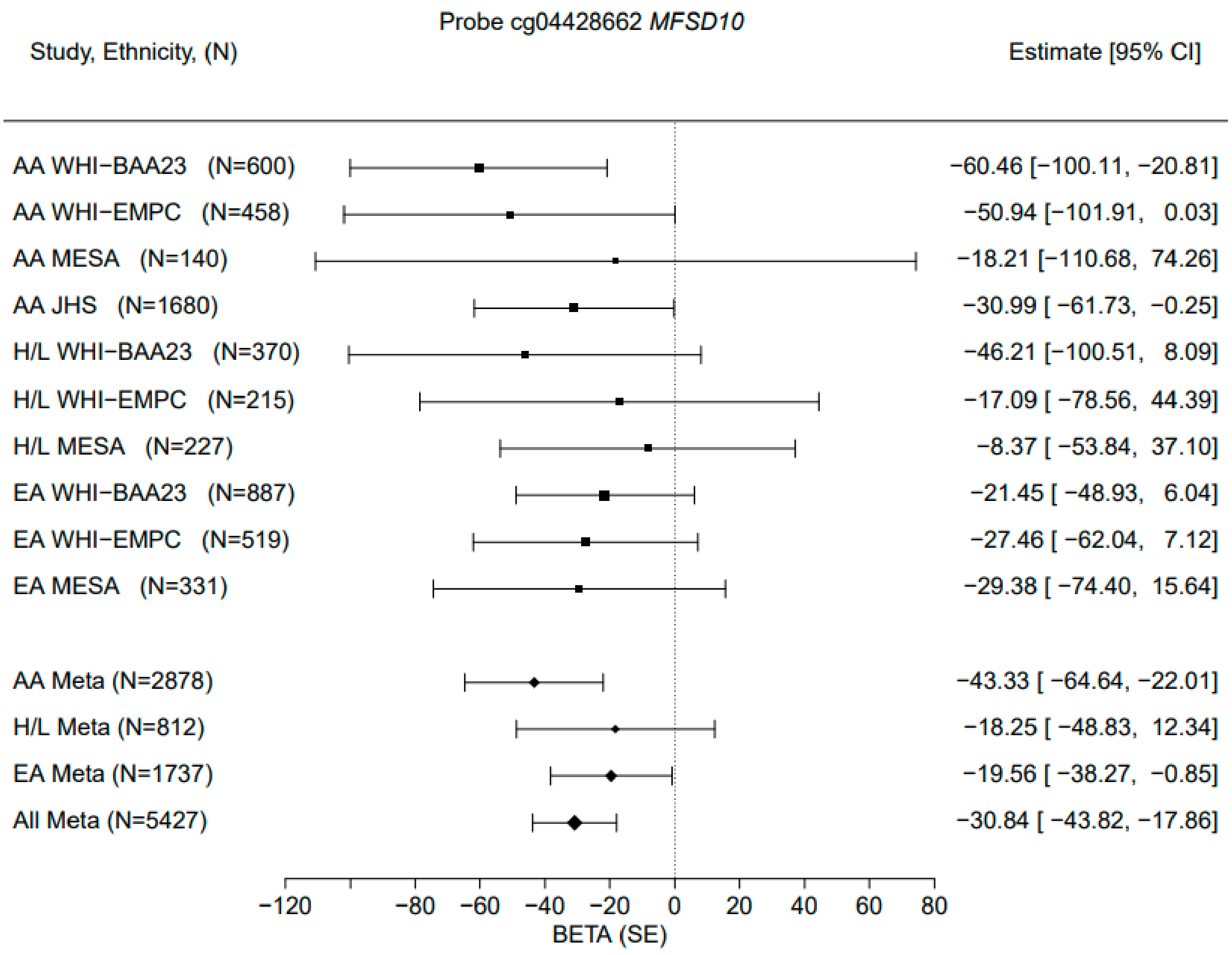

d. **Forest plot for probe cg23174201**

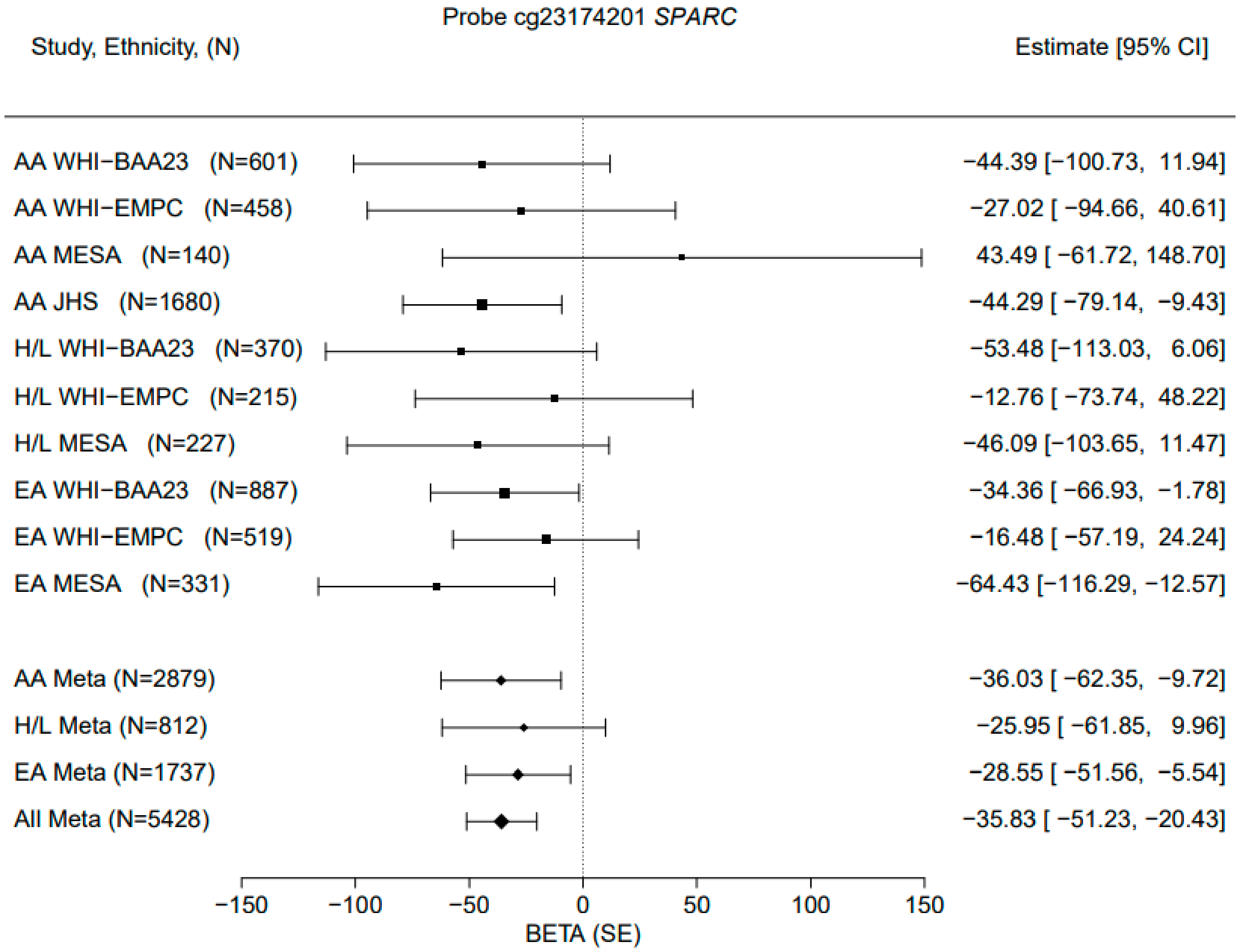

e. **Forest plot for probe cg17170437**

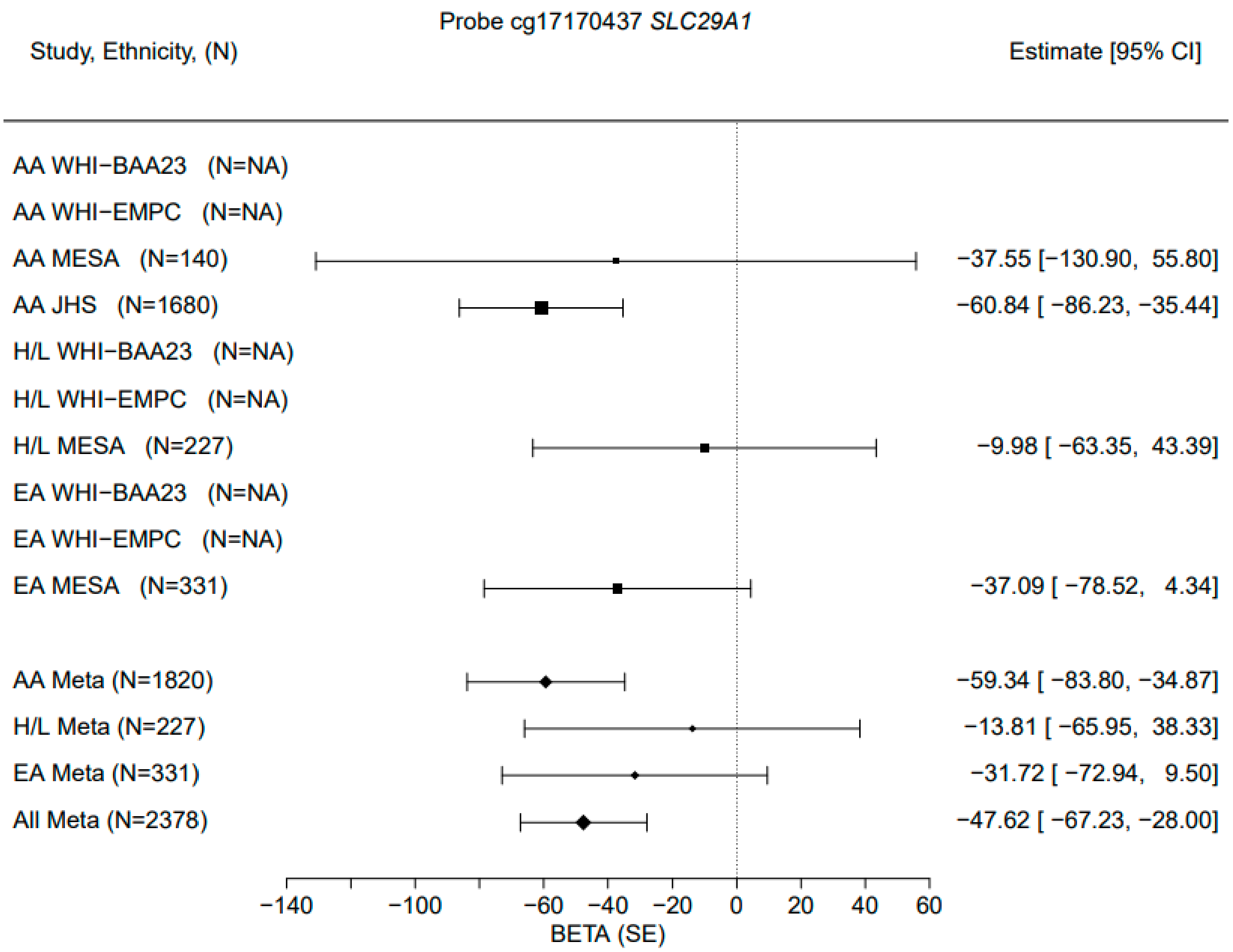

f. **Forest plot for probe cg14871770**

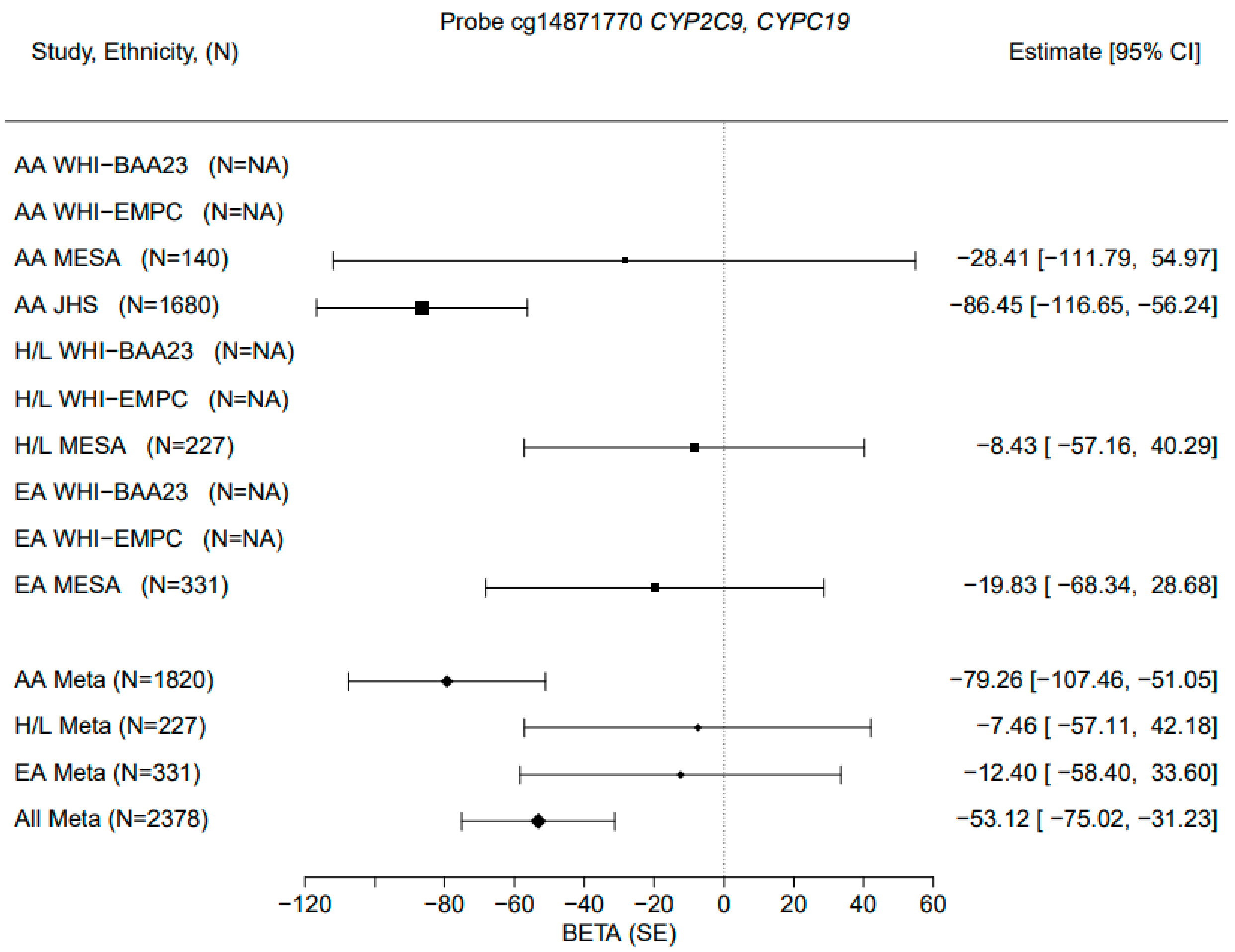

g. **Forest plot for probe cg02157636**

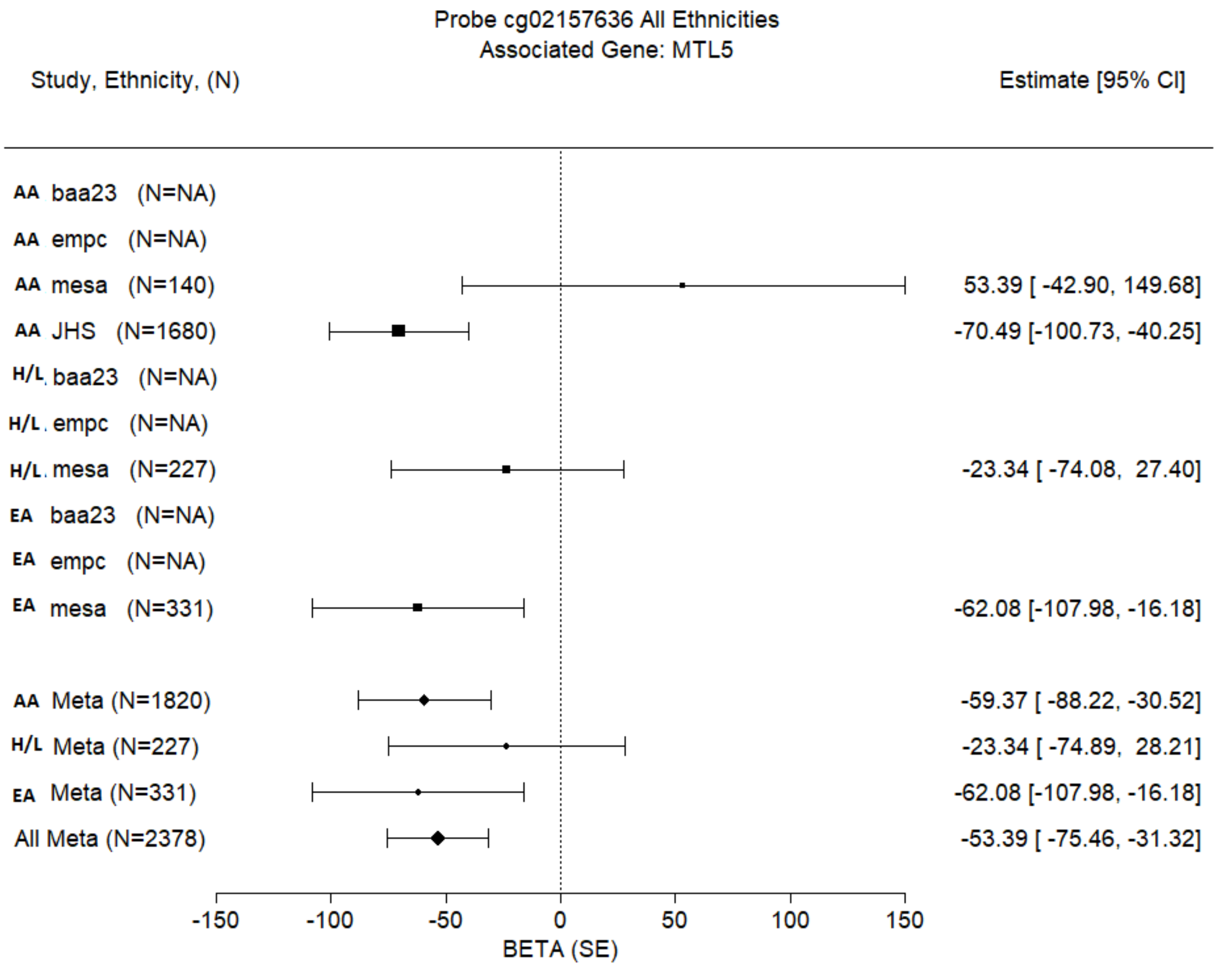

h. **Forest plot for probe cg26039141**

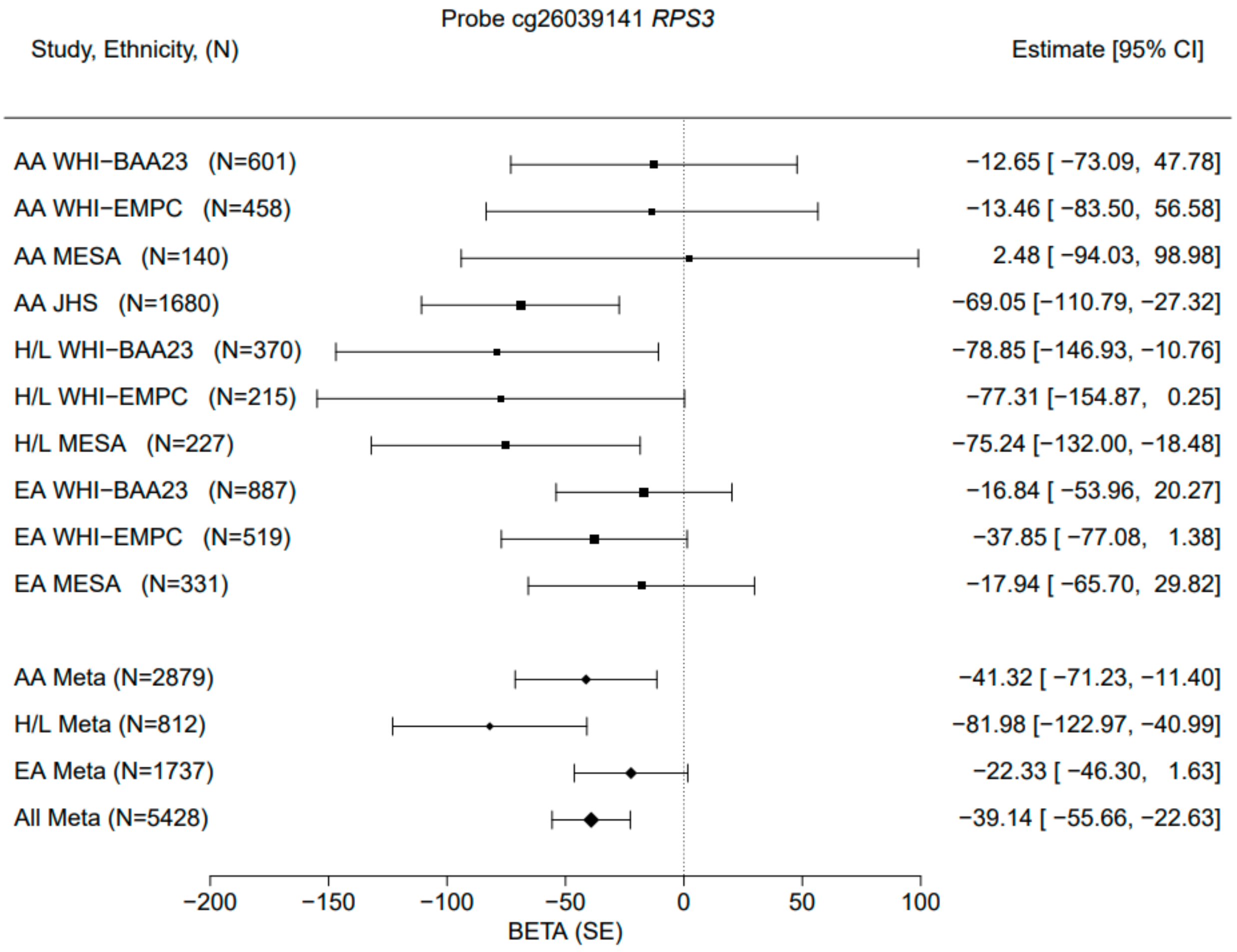

i. **Forest plot for probe cg22593432**

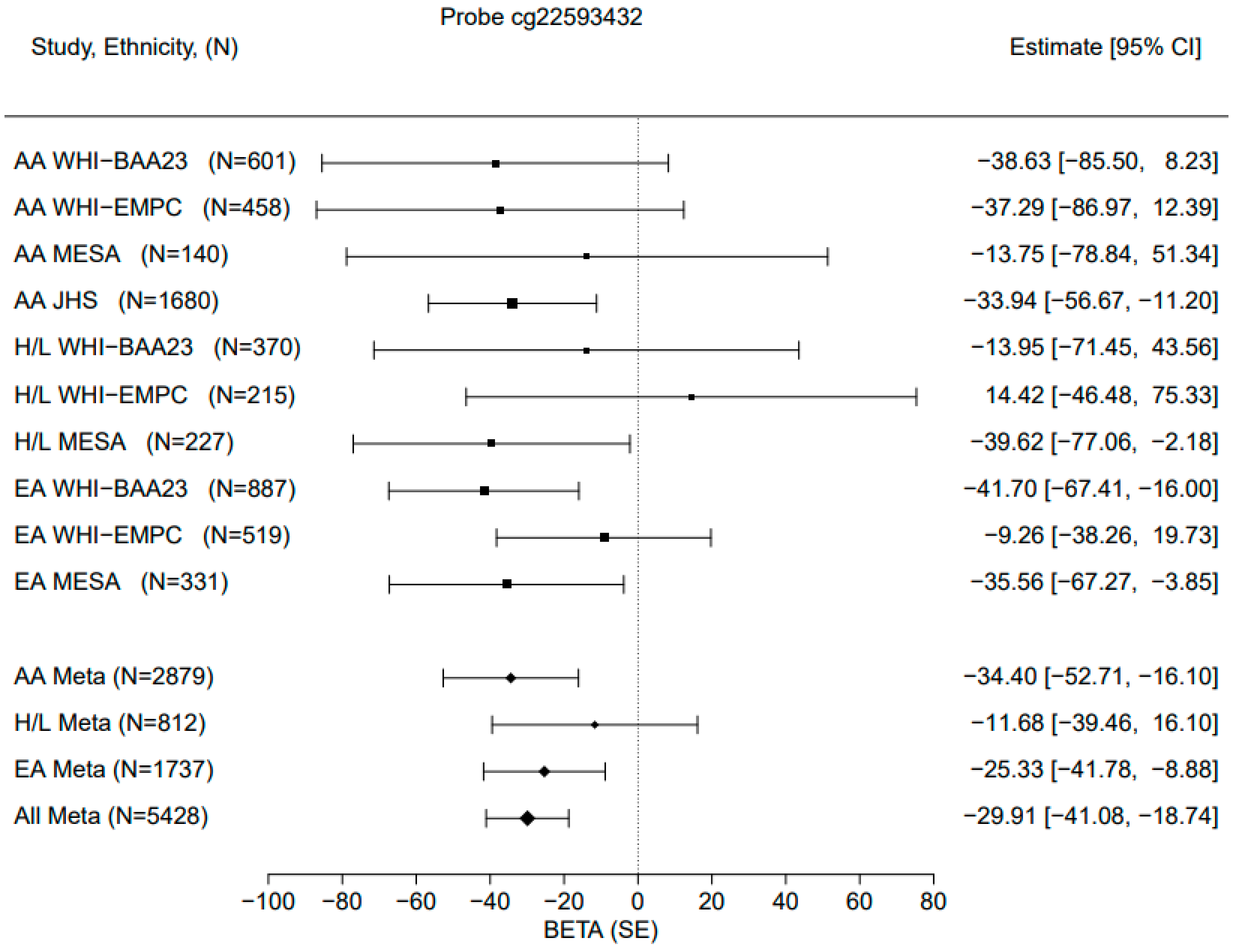

j. **Forest plot for probe cg11789371**

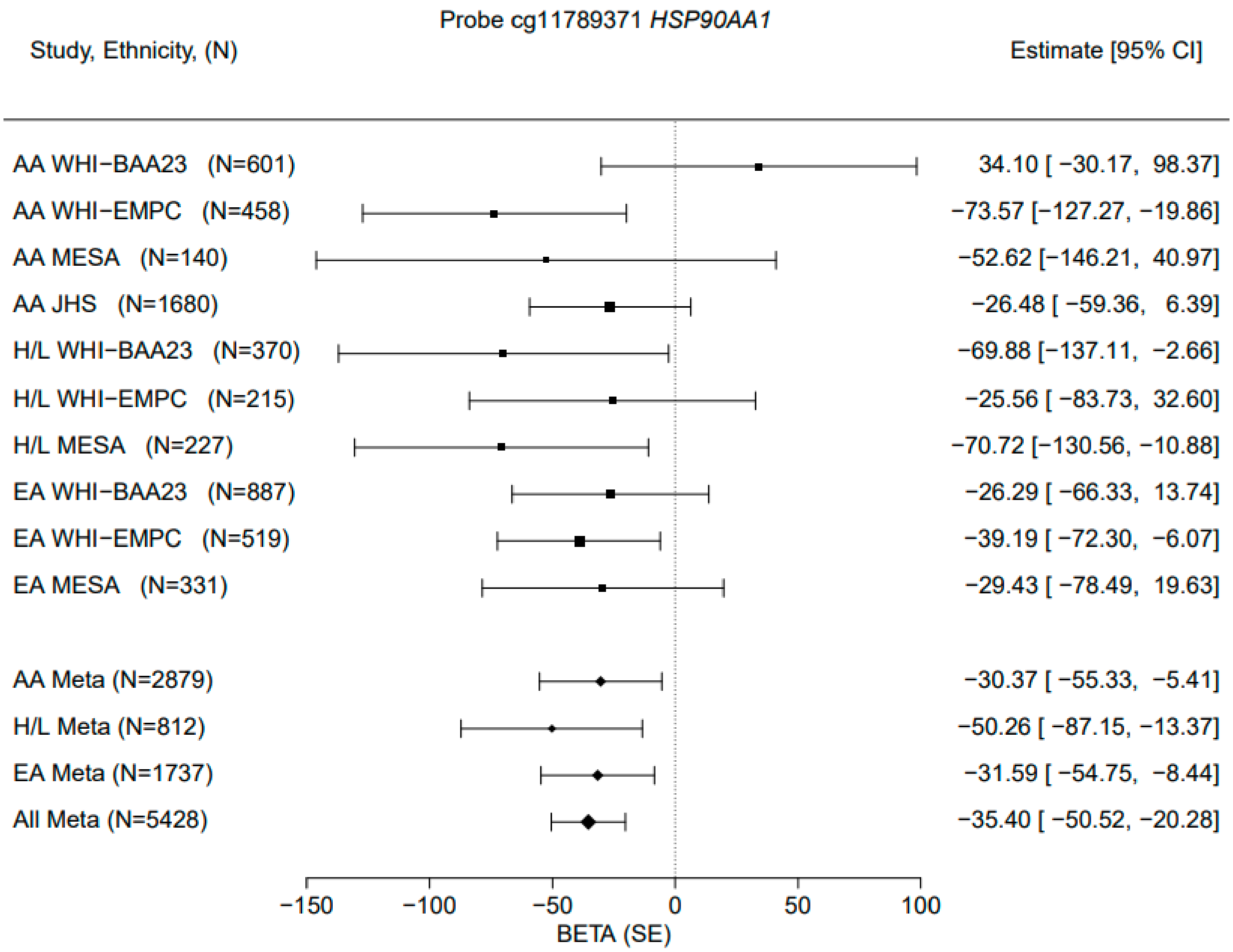

k. **Forest plot for probe cg05796561**

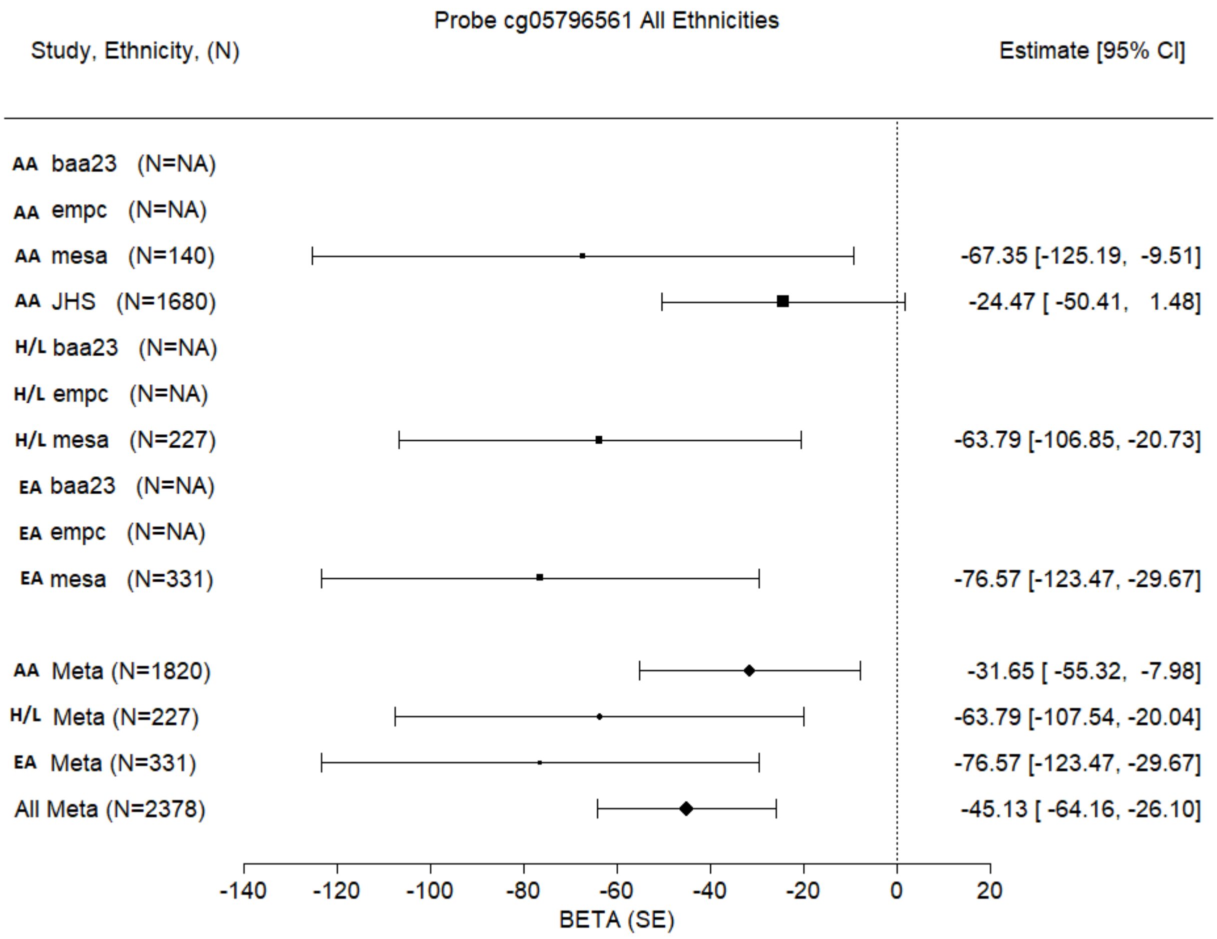

l. **Forest plot for probe cg17949885**

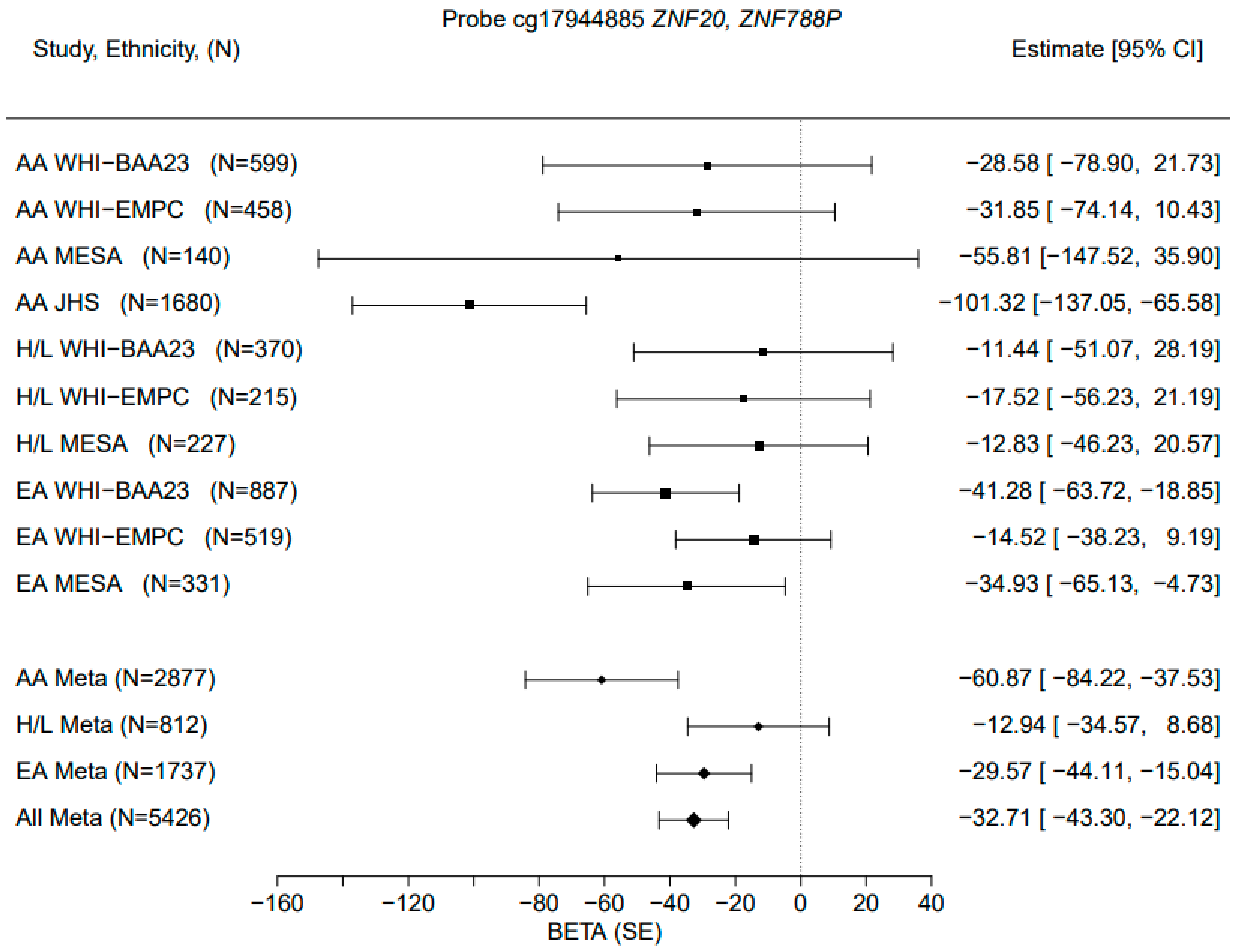

m. **Forest plot for probe cg15787712**

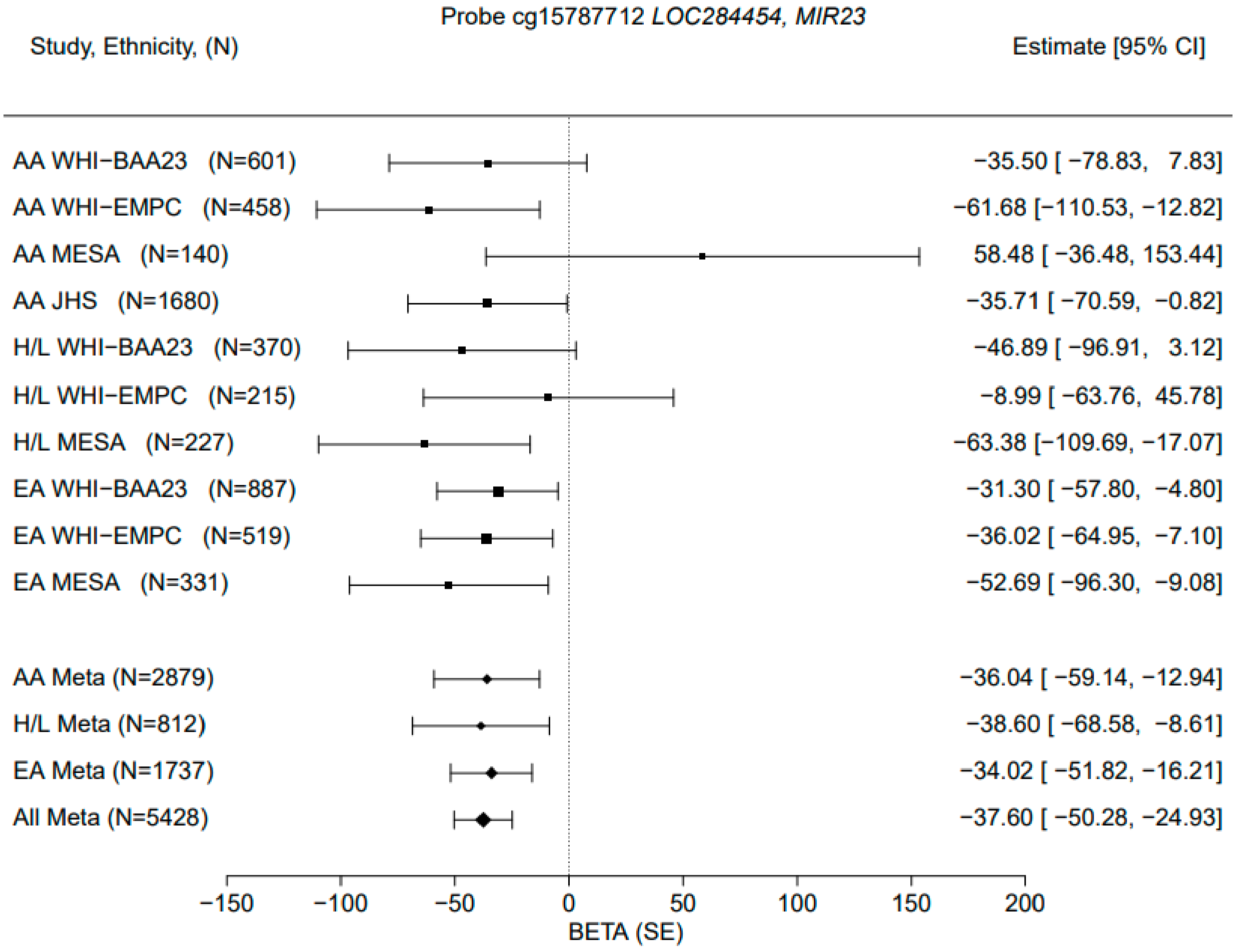

Figure S3. (a-m): Forest plots for DMPs showing the association with eGFR for each ethnicity/study sample, overall meta-analyses, and meta-analyses within each ethnicity for the discovery samples. Beta estimates are differences in DMPs per unit of eGFR. NA= not available. Genome-wide methylation data was obtained using Illumina 450K array for WHI-BAA23 and WHI-EMPC (AA, EA and H/L) and Illumina EPIC 850K array for MESA and JHS.

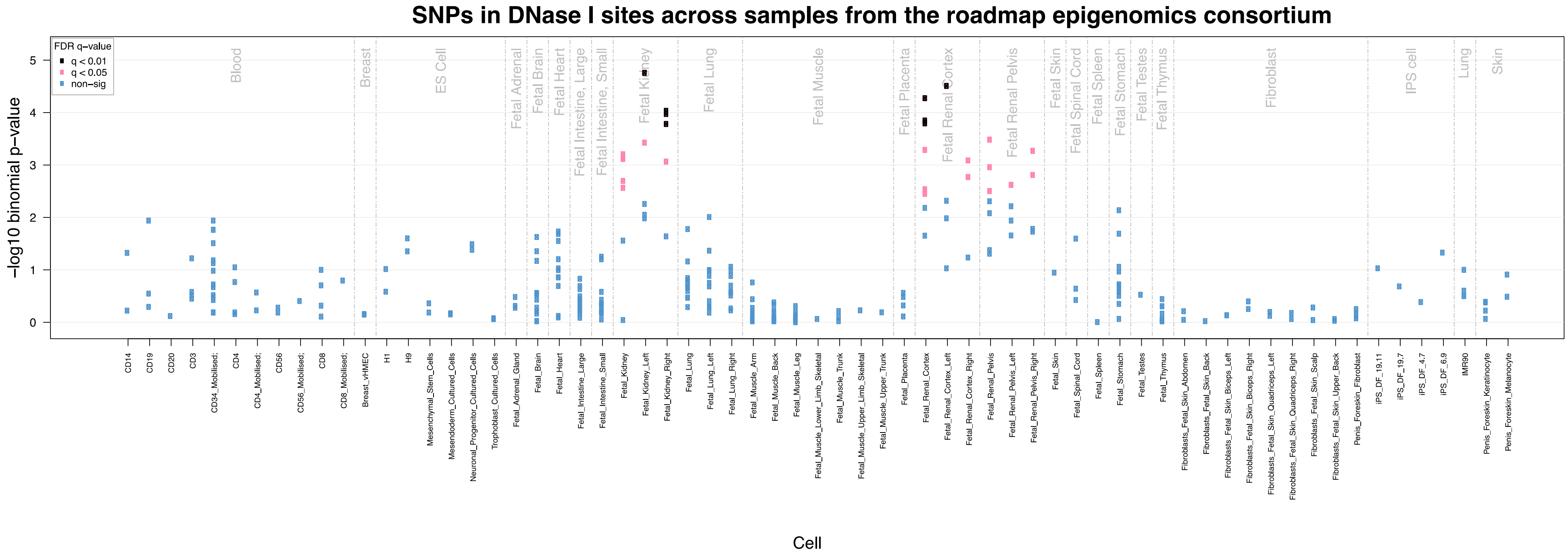

Figure S4. FORGE2 analysis for top eGFR COGENT GWAS SNPs: X axis indicates tissues/cell type samples used in the analysis; y axis shows FORGE2 enrichment (-log10 q-value) of the SNP set with DNase I hotspots for a range of tissue samples (significant samples in black). The highest ranked sample set (highest black points) shows the most significant enrichment is for kidney samples, which are highly ranked for the genome-wide significant set of SNPs associated with eGFR.

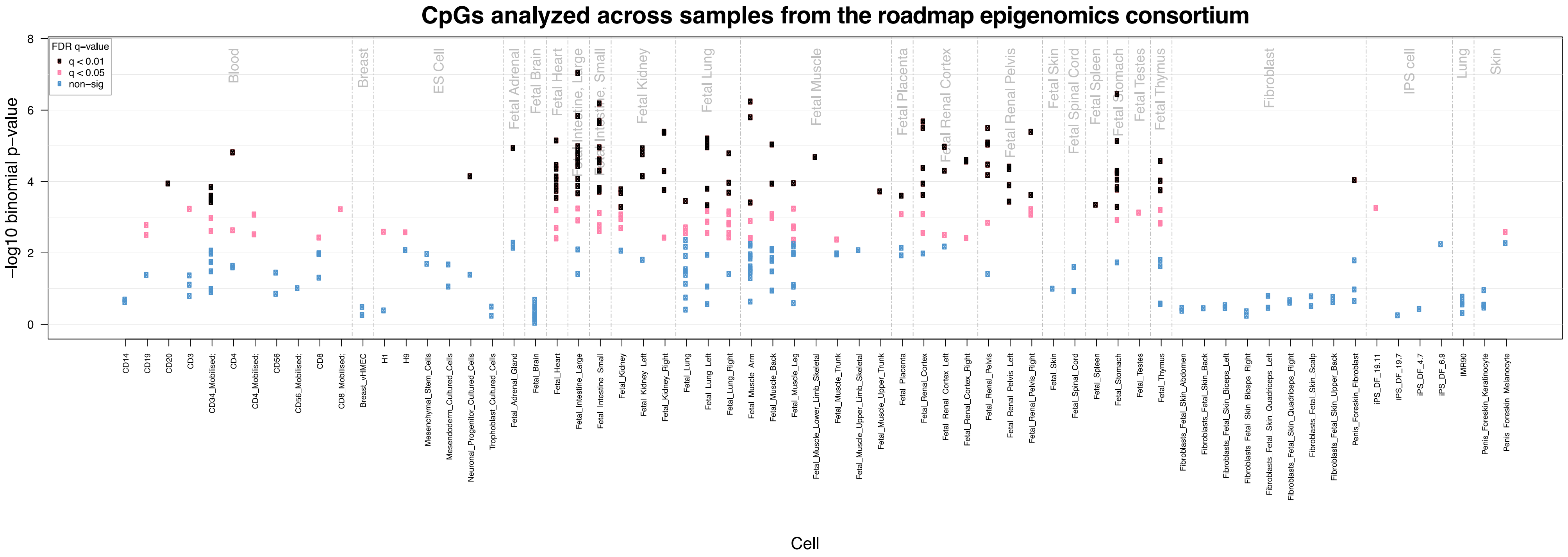

Figure S5. eFORGE analysis for top eGFR GWAS SNP-associated ARIES blood mQTL CpGs: X axis indicates tissues/cell type samples used in the analysis; y axis shows eFORGE enrichment (-log10 q-value) of the CpG set with DNase I hotspots for a range of tissue samples (significant samples in black). Results from this eFORGE analysis of significant ARIES mQTL CpGs associated with eGFR GWAS SNPs, indicate a higher-than expected overlap across DNase-seq hotspots from a range of tissues, including kidney, renal cortex and renal pelvis, among others.

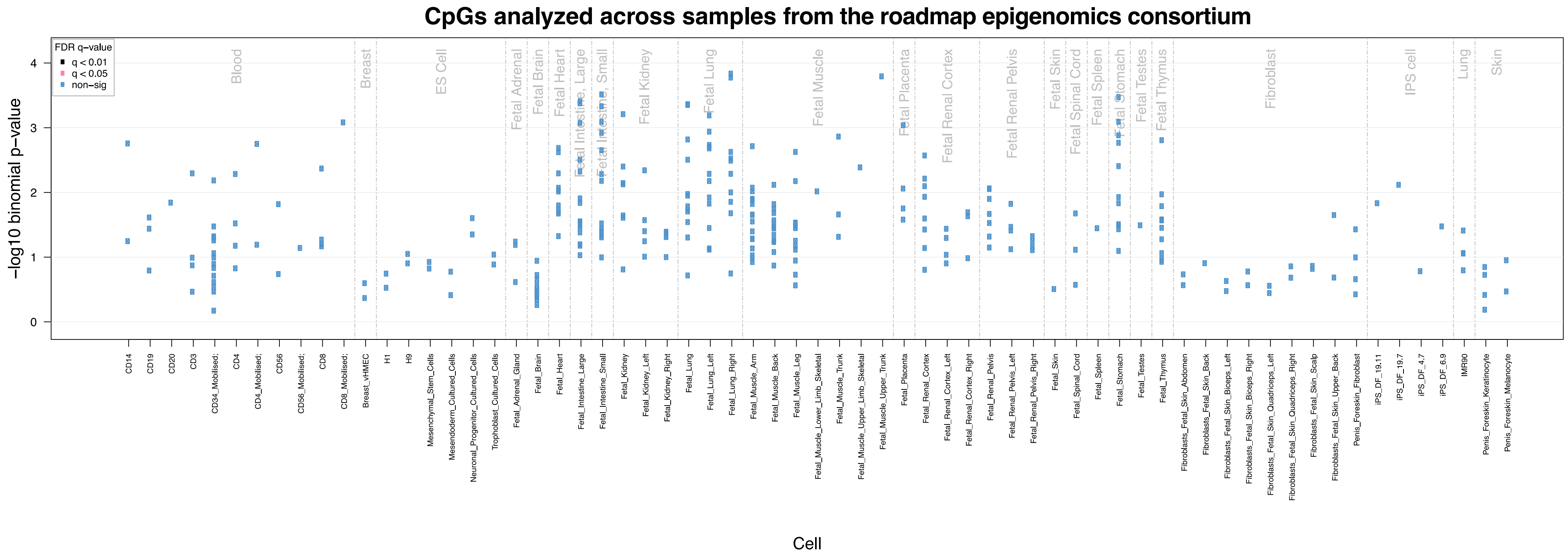

Figure S6. eFORGE analysis for a non-associated (p-value>0.95) kidney mQTL CpG set for top eGFR GWAS SNPs: X axis indicates tissues/cell type samples used in the analysis; y axis shows eFORGE enrichment (-log10 q-value) of the CpG set with DNase I hotspots for a range of tissue samples (non-significant samples in blue). Results from this eFORGE analysis of kidney mQTL CpG that are not significantly associated with eGFR GWAS SNPs indicate no enrichment across DNase-seq hotspots from a range of tissues, including kidney, renal cortex and renal pelvis, among others.

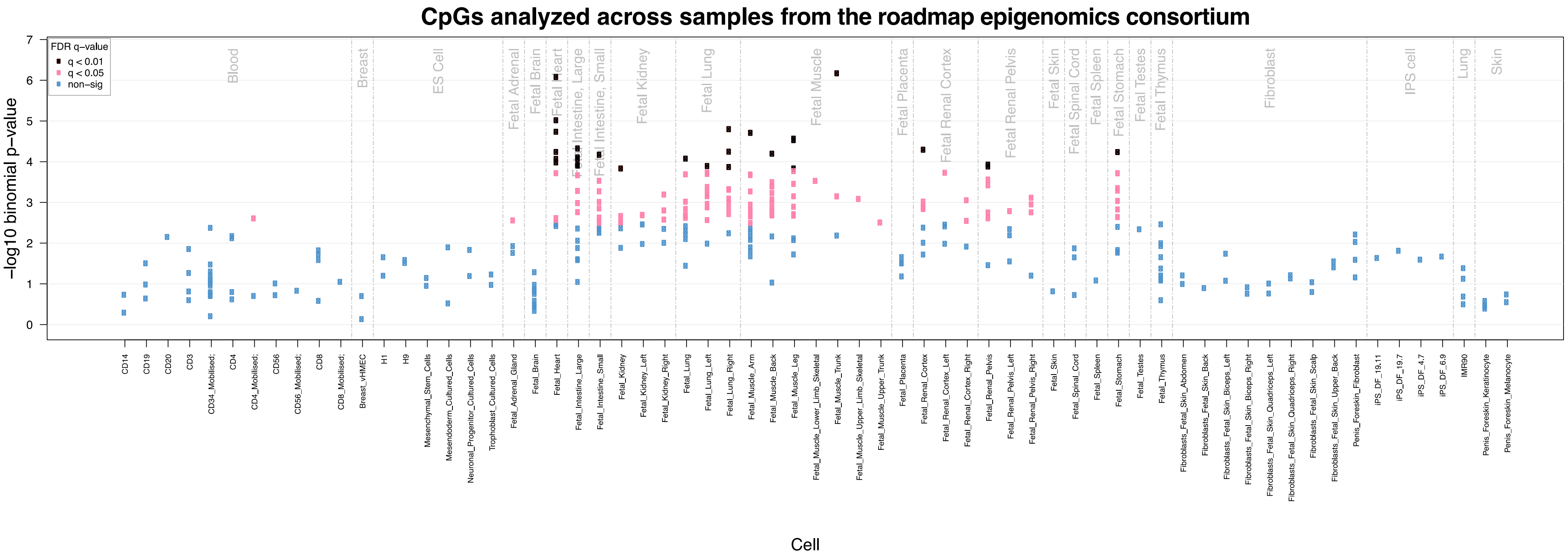

Figure S7. eFORGE analysis for top eGFR GWAS SNP-associated kidney mQTL CpGs (p-value<0.05): X axis indicates tissues/cell type samples used in the analysis; y axis shows eFORGE enrichment (-log10 q-value) of the CpG set with DNase I hotspots for a range of tissue samples (significant samples in black). Results from this eFORGE analysis of significant kidney mQTL CpGs associated with eGFR GWAS SNPs, indicate a higher-than expected overlap across DNase-seq hotspots from a range of tissues, including kidney, renal cortex and renal pelvis, among others.
