# Supplementary material for: Epigenome-wide association study of kidney function identifies trans-ethnic and ethnic-specific loci
Source: Genome Med. 2021 Apr 30;13:74. doi: 10.1186/s13073-021-00877-z (PMC8088054; doi:10.1186/s13073-021-00877-z)
Supplement: Supplementary file 2 — Additional file 2: Supplementary Methods. Description of different constituent cohorts and studies, in addition to power analyses. [file 13073_2021_877_MOESM2_ESM.docx]

**Additional File 2: Supplementary methods**

**Epigenome-wide association study of kidney function identifies shared and ethnic-specific loci**

**Supplementary Methods**

This section briefly describes the study design of all studies, and the phenotypic and DNA methylation (DNAm) data. Additional information can be found in Tables S1 and S2. The procedures used for DNA sample processing, assays and quality control of DNAm data can be found in the references. We provided more details on the MESA data given these have been recently generated and not previously published.

**Discovery studies**

**WHI** is a study of postmenopausal women (aged 50-79 years), comprising 161,808 women recruited from 40 U.S. clinical centers who participated in an observational study or in clinical trials during 1993-1998 as previously described [1-4]. DNAm was measured from whole blood using Illumina HumanMethylation450 (450 K) BeadChip array in 3,927 WHI participants from two studies: the Broad Agency Award 23 (WHI-BAA23), a case-control study of cardiovascular disease, and the Epigenetic Mechanisms of PM-Mediated Cardiovascular Disease Risk (WHI-EMPC), a stratified, random sample (n = 2,200) of participants who were examined between 1993 and 2001. In total, we took 3,050 participants forward for analysis (EA, AA, H/L) with complete data on DNA methylation, kidney traits and covariates.

**MESA** is a multi-ethnic study of subclinical cardiovascular disease and risk factors for cardiovascular disease [5], consisting of 6,814 asymptomatic men and women aged 45-84 (38% EA, 28% AA, 22% H/L, and 12% Asian, predominantly of Chinese descent) examined in 2000-2002, followed by four examination periods. Participants were recruited from six field centers across the United States: Wake Forest University, Columbia University, Johns Hopkins University, University of Minnesota, Northwestern University and University of California - Los Angeles. A subset of MESA participants was selected for multi-omics projects in the NHLBI Trans-Omic for Precision Medicine (TOPMed) program and were assayed using the Illumina EPIC 850K array. This study included 698 participants with DNAm measures and complete data on phenotypes and covariates. Since the MESA DNAm profiling has not been previously published, we include further details here.

1. MESA Genomic DNA Methylation Profiling Using Illumina EPIC Arrays

MESA study genomic methylation profiling was performed in the Methylation Characterization Center Laboratory at the University of Southern California (USC) under the aegis of the NHLBI TOPMed Program, MESA Multi-Omics Program. Laboratory standard operating procedure version NHLBI.2018.10.008 was used.

1.1 MESA Genomic DNA Conversion

Genomic DNA was shipped on dry ice to USC for Illumina EPIC array methylation profiling. On receipt, samples were quantitated using Nanodrop, and 600ng aliquots were bisulfite-converted using Zymo EZ methylation kits. Conversion QC was performed using published methods [6]. Four QC assays were used to measure conversion completeness and yield of converted DNA: ALU-C4 Ct value <19; CONV-100% <35; CONV-50% >40; and CONV-0% > 40; samples that failed any one of the four were re-tested and conversion repeated with a new DNA aliquot if necessary.

1.2 MESA Illumina Methylation EPIC Array Processing

Samples were randomized in batches of 192 (2 x 96) for array processing. Following vendor protocols, each batch of converted DNA samples was amplified, fragmented, precipitated, resuspended, and hybridized to the array. After the initial scan of each beadchip array was completed, the data was processed in the Illumina Genome Studio Methylation module with background correction and normalization to output preliminary beta values. Any sample with a preliminary call rate of <98% resulted in rescanning of the beadchip. If rescanning 2 times (of 3 total scans) did not yield all samples above 98% call rate, the low call rate samples were repeated using reserved sample from the precipitation step stored at -40degC (a 'rehyb'). For all passing samples, the best call rate scan was retained as raw data. Rehyb samples were rearrayed, and reprocessed as above. Of 1896 total MESA samples, 1662 (87.7%) were successful after the first 'hyb' (hybridization), and 1887 (99.5%) after two hybs, with excellent overall data quality.

1.3 MESA Methylation Quality Control and Normalization

The raw data was processed using R and the minfi package. Probes that were not significant above background signal were filtered at p-value > 0.05. Single sample Noob (ssNoob) was used for normalization [7] to derive corrected methylated and unmethylated signals, and thence the beta values, which are identical to the original Noob values on the beta scale [8].

1.4 Post-Normalization QC

Across all race/ethnic groups, samples were dropped based on non-concordance of EPIC SNPs (n=1) and related pairs (n=5). A further 6 samples were dropped as outliers by visual inspection of the first 10 PCs from within-race group methylation PCA. A total of 108,714 probes were filtered based on the recommendations of Zhou et al [9]. These included probes that had poor genomic mapping quality; a SNP present in the extension base causing color channel switch; non-unique 30 bp flanking sequence; extension base inconsistent with specified color channel; a SNP or SNV within 5 bp of the methylation base (<https://zwdzwd.github.io/InfiniumAnnotation>).

MESA WGS for the Trans-Omics in Precision Medicine (TOPMed) program was supported by the National Heart, Lung and Blood Institute (NHLBI). WGS for “NHLBI TOPMed: Multi-Ethnic Study of Atherosclerosis (MESA)” (phs001416.v1.p1) was performed at the Broad Institute of MIT and Harvard (3U54HG003067-13S1). Centralized read mapping and genotype calling, along with variant quality metrics and filtering were provided by the TOPMed Informatics Research Center (3R01HL-117626-02S1). Phenotype harmonization, data management, sample-identity QC, and general study coordination were provided by the TOPMed Data Coordinating Center (3R01HL-120393-02S1).

**JHS** is a study of cardiovascular disease and its risk factors in AA, comprising 5,306 African Americans aged 21 to 94 years recruited from the Jackson, MS metropolitan area from 2000-04, with four follow-up exams. Family members were recruited to create a nested family cohort (22% of the sample) [10]. A total of 3,404 consenting JHS participants had genomic DNA extracted from leukocytes, and genome-wide genotyping [11]. DNAm was measured using the Illumina EPIC 850K array in 1,680 AA participants at visit 1 that had kidney biomarkers and clinical data.

**Replication studies.**

**CATHeterization GENetics** (CATHGEN) is a biorepository of clinical samples from a prospectively collected clinical cohort of individuals undergoing cardiac catheterization at Duke University [12]. Blood samples were obtained during catheterization in consented participants, which linked to clinical and exposure data including laboratory data. Blood-based DNAm was obtained using the Illumina EPIC array [13]. Only participants with available serum creatinine were included in this study (n= 547, 344 EA and 212 AA). 9 participants on hemodialysis were excluded.

**Generation Scotland** (GS) is a family-based and population-based study consisting of 23,690 EA participants recruited from general medical practices across Scotland between 2006-2011 [14]. Probands aged between 35 and 65 years were asked to invite first-degree relatives to join the study, for a final size of 24,090. A variety of cognitive, physical, and health data were collected at the study baseline along with blood or saliva samples for DNA genotyping. Blood-based DNAm data were obtained using the Illumina EPIC array on a subset of 5,200 participants, of whom 2,586 unrelated individuals were included as set 1 (ages: 18-87 years) [15]. The second set of blood-based DNAm data included 4,450 individuals who were unrelated to each other (genetic relatedness < 0.05) and were also unrelated to participants from the first GS DNAm dataset.

**HyperGEN**. The HyperGEN study is one of the four networks in the Family Blood Pressure Program (FBPP) supported by the National Heart, Lung, and Blood Institute to identify genetic contributors to hypertension, and target end organ damage related to hypertension [16]. HyperGEN is a family-based study with a sib-pair design. Hypertensive African American sibships were recruited from Forsyth County, NC and from the community-at-large in Birmingham, AL from 1995 to 2000. Sib-pairs with hypertension onset before age 60 were recruited in the first phase. The study was subsequently extended to other siblings and the offspring of the hypertensive probands who were unmedicated adults. Hypertension was defined as having an average systolic blood pressure ≥ 140 mmHg and/or average diastolic blood pressure ≥ 90 mmHg at two separate clinic visits or taking any antihypertensive medication. An ancillary epigenetic study of left ventricular hypertrophy was conducted comprising participants included in this study [17]. DNA (500 ng) extracted from buffy coat was hybridized to the Illumina Infinium HumanMethylation450 (450K) BeadChip array (Illumina Inc, San Diego, CA) to assess methylation at cytosine-phosphate-guanine (CpG) sites. Analysis of the intensity files with Illumina GenomeStudio generated beta (β) scores of the proportion of signal of the methylation probe in the sample, and respective detection p-values. Quality control (QC) procedures removed any CpG probe where more than 10% of samples failed to yield adequate intensity. After these QC filters, there were 485,512 CpG sites eligible for analysis. For correction of systematic technical biases in the 450K assay, normalization was performed using the Quantile-Quantile method using the R package *minfi*. Cell count proportions (CD8+ T lymphocytes, CD4+ T lymphocytes, natural killer cells, B cells, monocytes, and granulocytes) were generated using the algorithm developed by Houseman *et al.*, which predicts the underlying cellular composition of each sample from DNAm patterns. Ancestry principal components (PCs) were generated from whole genome sequencing data as described.

**meQTL in normal kidney tissue**. Genotypes in non-TCGA studies (TRANSLATE, TRANSLATE-T, RESPOND and REPAIR) were measured using Infinium HumanCoreExome-24 BeadChip arrays and the allele calls were made using Genome Studio. For TCGA data, genotyping was conducted using the Affymetrix Genome-Wide Human SNP Array 6.0 and alleles were called using Birdsuite software [18-20]. Samples were excluded if their genotyping rate was < 95%, their heterozygosity rate was outside ± 3 standard deviations from the mean, they had cryptic relatedness with other individuals, were of non-white European genetic ancestry or had discordant sex information (inconsistency between declared and genotyped sex). Genetic variants were excluded if their genotyping rate was < 95%, they mapped to the Y chromosome or mitochondrial DNA, had ambiguous chromosomal location, violated Hardy-Weinberg equilibrium (P < 0.001) or if their minor allele frequency (MAF) was < 5%. Genotype imputation was conducted on the Michigan Imputation Server using minimac3 with Haplotype Reference Consortium data as the reference panel.

DNA was extracted from homogenized renal tissue samples. Non-TCGA kidney DNA samples underwent bisulfite conversion and were hybridized with the Illumina HumanMethylation450 BeadChip array or Methylation EPIC BeadChip array as per the manufacturer’s guidelines. All TCGA kidney DNA samples were processed with HumanMethylation450 BeadChip arrays. We downloaded DNAm data from the GDC legacy portal (https://portal.gdc.cancer.gov/) using the following criteria: project name – TCGA, primary site – kidney, sample type – solid tissue normal, race – white, ethnicity – not Hispanic or Latino, data category – raw microarray data, data type – raw intensities, experimental strategy – methylation array, data format – idat, platform – Illumina Human Methylation 450. The data from all studies were merged using R package minfi. M-values were normalized using the “dasen” method from the wateRmelon R package, and standard quality control for samples and probes was applied. The final combined dataset consisted of 195 samples: 177 from non-TCGA studies and 18 from TCGA. In total, 374,826 CpG sites were available for further analyses after quality control filters.

**Power analyses**

We performed power calculations to determine power for DMPs associated with each discovery sample based on the number of CpG tested using the tool from Mansell *et al.*, 2018, <https://epigenetics.essex.ac.uk/shiny/EPICDNAmPowerCalcs/>, Power analysis figures 1-12, tables 1 and 2):

|  | AA | EA | H/L | Trans-ethnic |
| --- | --- | --- | --- | --- |
| Sample n | 2,879 | 1,737 | 812 | 5,428 |
| Array | 450K+EPIC 850K | 450K | 450K | 450K+EPIC 850K |
| CpG sites n | 656,213 | 364,117 | 364,117 | 655,786 |
| Significant CpGs identified in the discovery samples | 23 | 5 | 5 | 78 |
| Power to detect a 1% mean difference at significance threshold 1e-06 across all sites | >90% power for 71.8% sites | >90% power for 54.3% sites | >90% power for 26.6% sites | >90% power for 88.5% sites |
| Power to detect a 2% mean difference at significance threshold 1e-06 across all sites | >90% power for 97.7% sites | >90% power for 92.7% sites | >90% power for 75.2% sites | >90% power for 99.2% sites |

**Power analysis table 1: Number of identified sites and DNA methylation power calculations.** Note that only AA and trans-ethnic meta-analyses included EPIC 850K array probes as we required a minimum of two studies contributing to meta-analyses.


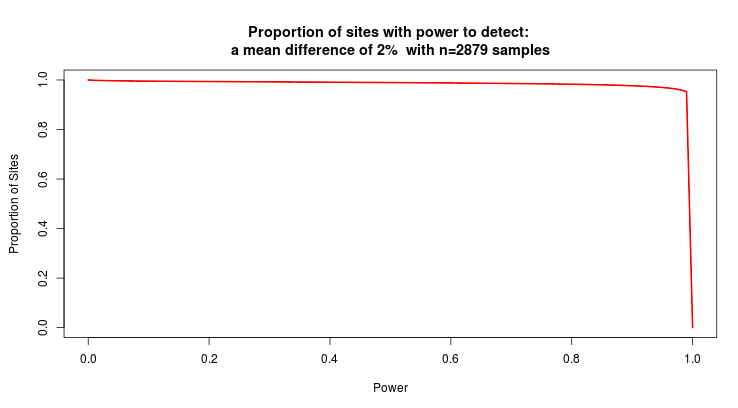

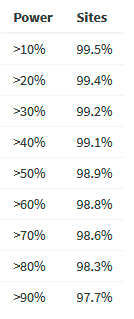


**Power analysis figure 1:** Power for AA (n=2879, 2%).


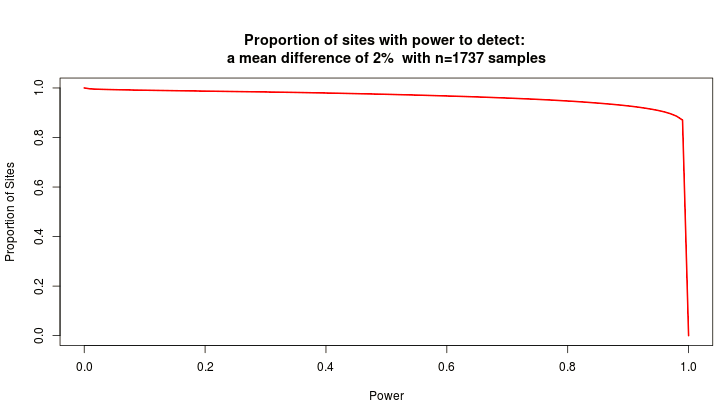

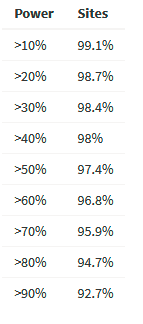


**Power analysis figure 2:** Power for EA (n=1737, 2%).


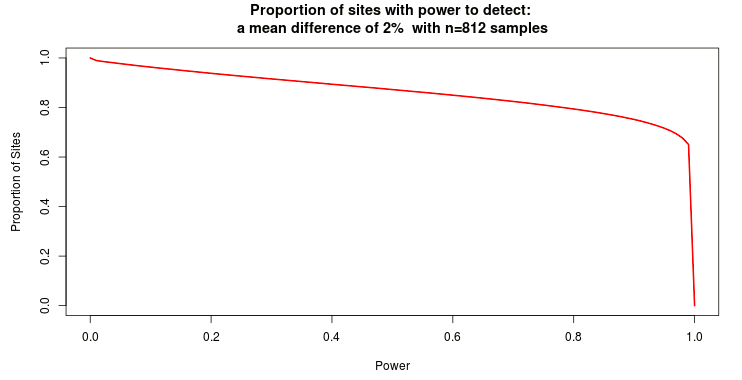

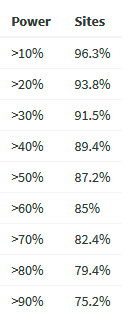


**Power analysis figure 3:** Power for H/L (n=812, 2%).


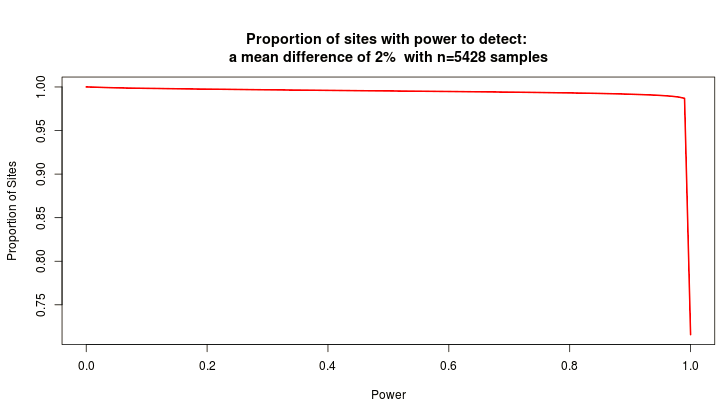

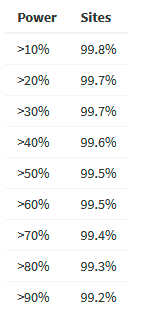


**Power analysis figure 4:** Power for trans-ethnic (n=5428, 2%).

*
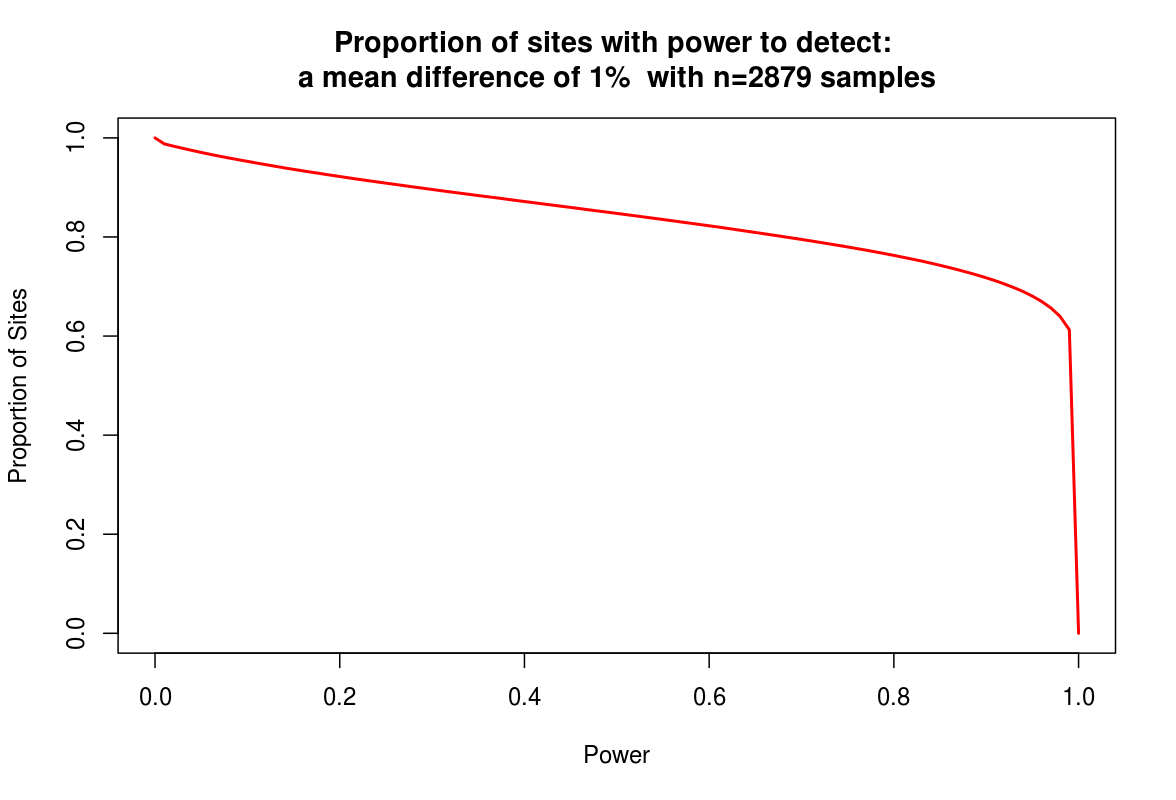

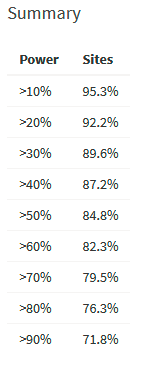
*

**Power analysis figure 5:** Power for AA (n=2879, 1%).

*
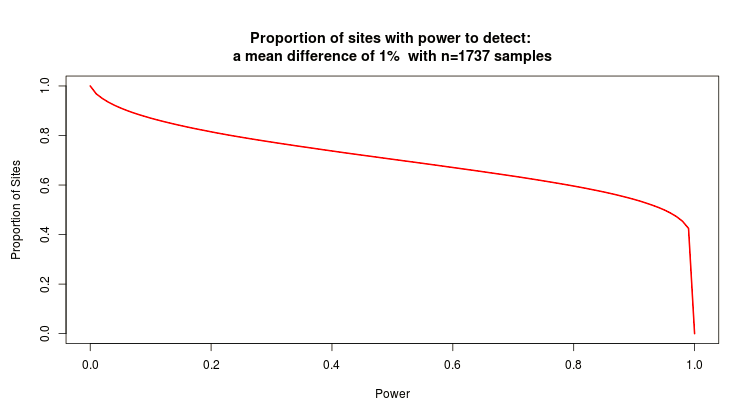
***
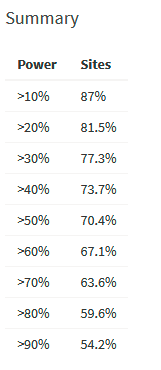
**

**Power analysis figure 6:** Power for EA (n=1737, 1%).

**
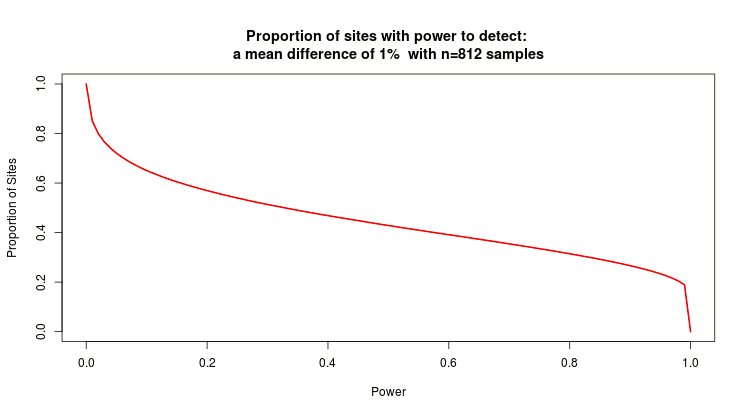

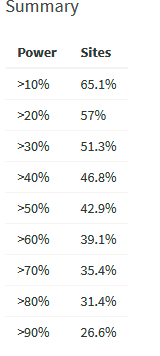
**

**Power analysis figure 7:** Power for H/L (n=812, 1%).

**
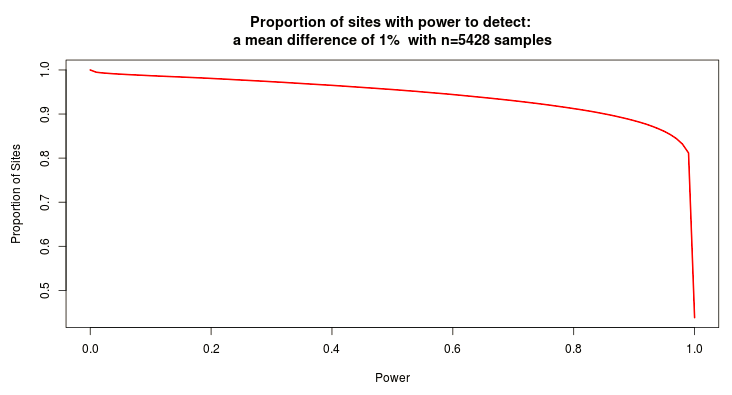

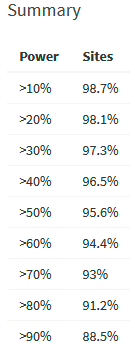
**

**Power analysis figure 8:** Power for trans-ethnic (n=5428, 1%).

|  | AA | trans-ethnic |
| --- | --- | --- |
| Sample n | 1,820 | 2,378 |
| Power to detect a 1% mean difference at significance threshold 1e-06 across all sites | >90% power for 55% sites | >90% power for 65.4 % sites |
| Power to detect a 2% mean difference at significance threshold 1e-06 across all sites | >90% power for 93.4% sites | >90% power for 96.4% sites |

**Power analysis table 2:** Power analyses for EPIC-specific probes.

**
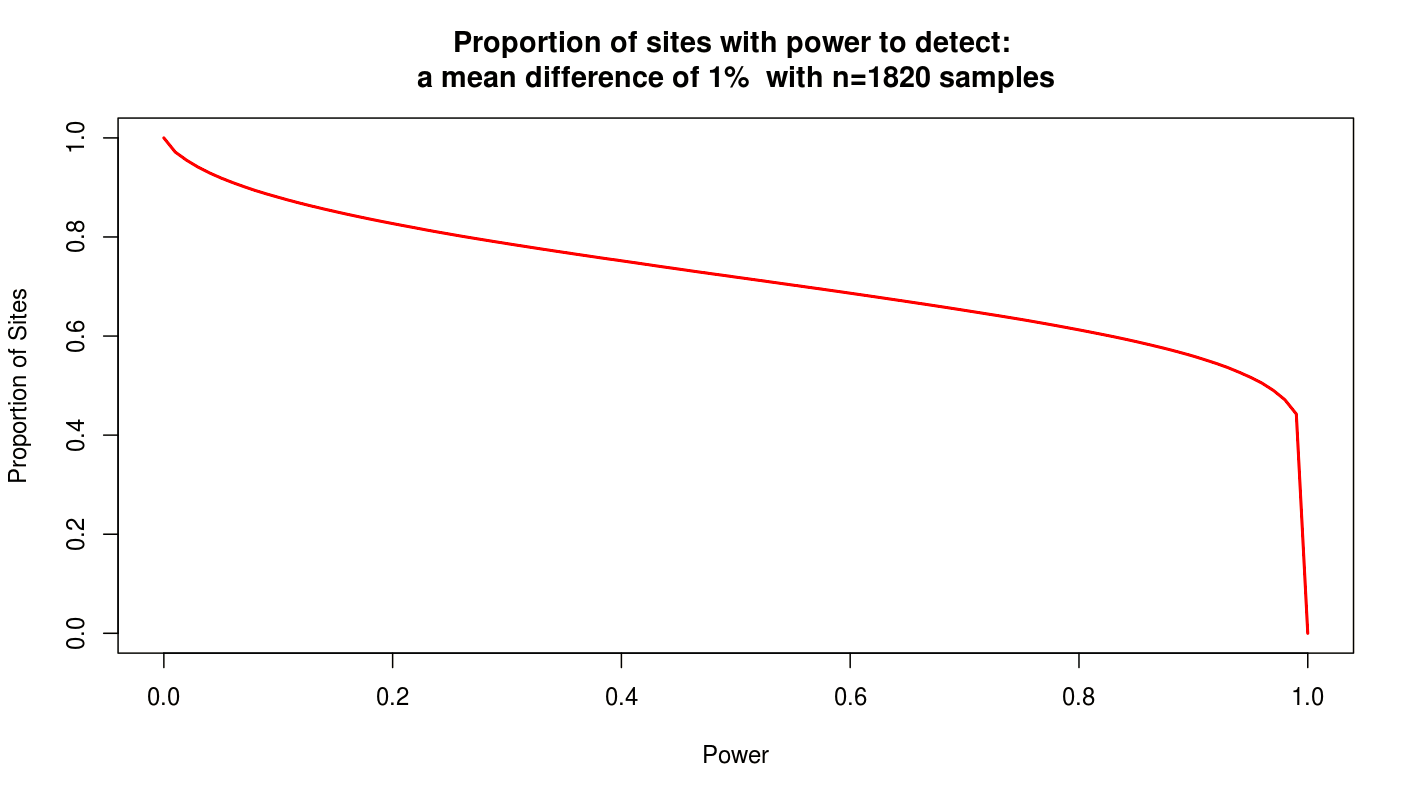

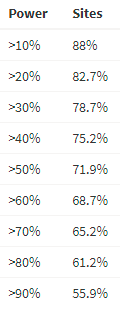
**

**Power analysis figure 9:** Power for EPIC-specific probes in AA (n=1820, 1%).

**
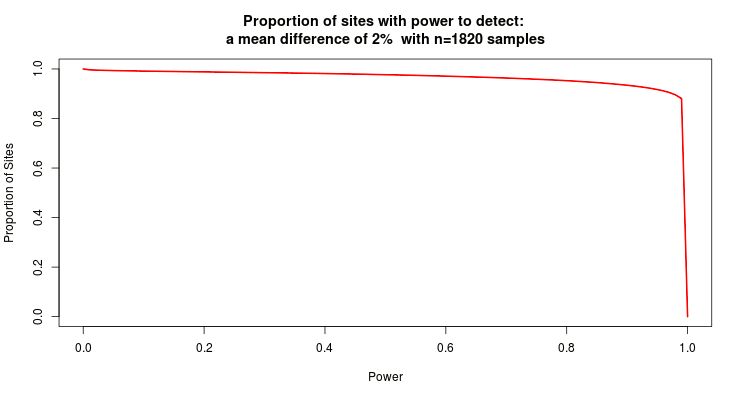

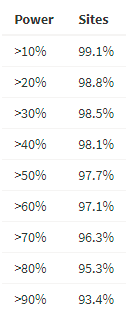
**

**Power analysis figure 10:** Power for EPIC-specific probes in AA (n=1820, 2%).

**
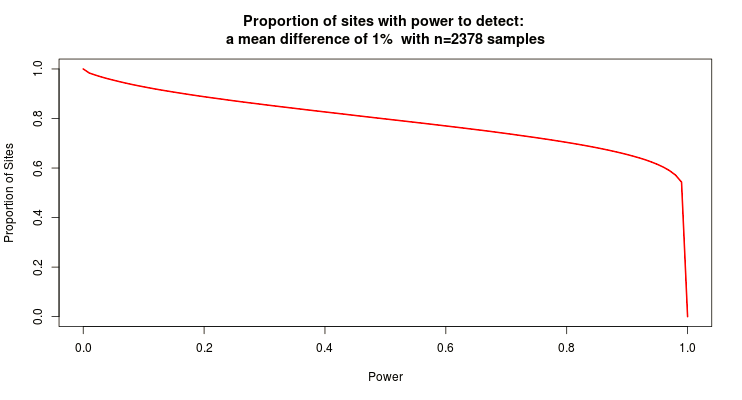

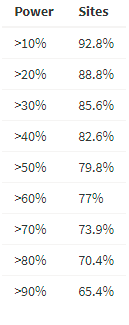
**

**Power analysis figure 11:** Power for EPIC-specific probes in trans-ethnic (n=2378, 1%).

**
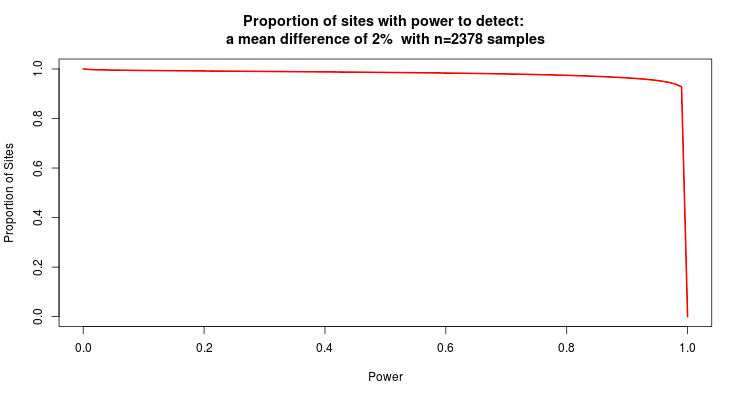

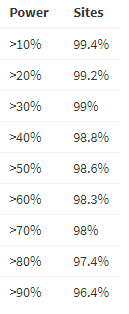
**

**Power analysis figure 12:** Power for EPIC-specific probes in trans-ethnic (n=2378, 2%).

**References**

1. **Design of the Women's Health Initiative clinical trial and observational study. The Women's Health Initiative Study Group.** *Control Clin Trials* 1998, **19:**61-109.

2. Anderson GL, Manson J, Wallace R, Lund B, Hall D, Davis S, Shumaker S, Wang CY, Stein E, Prentice RL: **Implementation of the Women's Health Initiative study design.** *Annals of epidemiology* 2003, **13:**S5-17.

3. Howard BV, Van Horn L, Hsia J, Manson JE, Stefanick ML, Wassertheil-Smoller S, Kuller LH, LaCroix AZ, Langer RD, Lasser NL, et al: **Low-fat dietary pattern and risk of cardiovascular disease: the Women's Health Initiative Randomized Controlled Dietary Modification Trial.** *JAMA : the journal of the American Medical Association* 2006, **295:**655-666.

4. Jackson RD, LaCroix AZ, Gass M, Wallace RB, Robbins J, Lewis CE, Bassford T, Beresford SA, Black HR, Blanchette P, et al: **Calcium plus vitamin D supplementation and the risk of fractures.** *The New England journal of medicine* 2006, **354:**669-683.

5. Bild DE, Bluemke DA, Burke GL, Detrano R, Diez Roux AV, Folsom AR, Greenland P, Jacob DR, Jr., Kronmal R, Liu K, et al: **Multi-Ethnic Study of Atherosclerosis: objectives and design.** *Am J Epidemiol* 2002, **156:**871-881.

6. Campan M, Weisenberger DJ, Trinh B, Laird PW: **MethyLight.** *Methods Mol Biol* 2009, **507:**325-337.

7. Fortin JP, Triche TJ, Jr., Hansen KD: **Preprocessing, normalization and integration of the Illumina HumanMethylationEPIC array with minfi.** *Bioinformatics* 2017, **33:**558-560.

8. Triche TJ, Jr., Weisenberger DJ, Van Den Berg D, Laird PW, Siegmund KD: **Low-level processing of Illumina Infinium DNA Methylation BeadArrays.** *Nucleic Acids Res* 2013, **41:**e90.

9. Zhou W, Laird PW, Shen H: **Comprehensive characterization, annotation and innovative use of Infinium DNA methylation BeadChip probes.** *Nucleic Acids Res* 2017, **45:**e22.

10. Taylor HA, Jr., Wilson JG, Jones DW, Sarpong DF, Srinivasan A, Garrison RJ, Nelson C, Wyatt SB: **Toward resolution of cardiovascular health disparities in African Americans: design and methods of the Jackson Heart Study.** *Ethnicity & disease* 2005, **15:**S6-4-17.

11. Wilson JG, Rotimi CN, Ekunwe L, Royal CD, Crump ME, Wyatt SB, Steffes MW, Adeyemo A, Zhou J, Taylor HA, Jr., Jaquish C: **Study design for genetic analysis in the Jackson Heart Study.** *Ethnicity & disease* 2005, **15:**S6-30-37.

12. Kraus WE, Granger CB, Sketch MH, Jr., Donahue MP, Ginsburg GS, Hauser ER, Haynes C, Newby LK, Hurdle M, Dowdy ZE, Shah SH: **A Guide for a Cardiovascular Genomics Biorepository: the CATHGEN Experience.** *J Cardiovasc Transl Res* 2015, **8:**449-457.

13. Abdulrahim JW, Kwee LC, Grass E, Siegler IC, Williams R, Karra R, Kraus WE, Gregory SG, Shah SH: **Epigenome-Wide Association Study for All-Cause Mortality in a Cardiovascular Cohort Identifies Differential Methylation in Castor Zinc Finger 1 (CASZ1).** *J Am Heart Assoc* 2019, **8:**e013228.

14. Smith BH, Campbell A, Linksted P, Fitzpatrick B, Jackson C, Kerr SM, Deary IJ, Macintyre DJ, Campbell H, McGilchrist M, et al: **Cohort Profile: Generation Scotland: Scottish Family Health Study (GS:SFHS). The study, its participants and their potential for genetic research on health and illness.** *Int J Epidemiol* 2013, **42:**689-700.

15. McCartney DL, Zhang F, Hillary RF, Zhang Q, Stevenson AJ, Walker RM, Bermingham ML, Boutin T, Morris SW, Campbell A, et al: **An epigenome-wide association study of sex-specific chronological ageing.** *Genome Med* 2019, **12:**1.

16. Williams RR, Rao DC, Ellison RC, Arnett DK, Heiss G, Oberman A, Eckfeldt JH, Leppert MF, Province MA, Mockrin SC, Hunt SC: **NHLBI family blood pressure program: methodology and recruitment in the HyperGEN network. Hypertension genetic epidemiology network.** *Ann Epidemiol* 2000, **10:**389-400.

17. Akinyemiju T, Do AN, Patki A, Aslibekyan S, Zhi D, Hidalgo B, Tiwari HK, Absher D, Geng X, Arnett DK, Irvin MR: **Epigenome-wide association study of metabolic syndrome in African-American adults.** *Clin Epigenetics* 2018, **10:**49.

18. Rowland J, Akbarov A, Eales J, Xu X, Dormer JP, Guo H, Denniff M, Jiang X, Ranjzad P, Nazgiewicz A, et al: **Uncovering genetic mechanisms of kidney aging through transcriptomics, genomics, and epigenomics.** *Kidney Int* 2019, **95:**624-635.

19. Tomaszewski M, Eales J, Denniff M, Myers S, Chew GS, Nelson CP, Christofidou P, Desai A, Busst C, Wojnar L, et al: **Renal Mechanisms of Association between Fibroblast Growth Factor 1 and Blood Pressure.** *J Am Soc Nephrol* 2015, **26:**3151-3160.

20. Xu X, Eales JM, Akbarov A, Guo H, Becker L, Talavera D, Ashraf F, Nawaz J, Pramanik S, Bowes J, et al: **Molecular insights into genome-wide association studies of chronic kidney disease-defining traits.** *Nat Commun* 2018, **9:**4800.
